# Supplementary material for: Multi-Channel 3D Deep Feature Learning for Survival Time Prediction of Brain Tumor Patients Using Multi-Modal Neuroimages
Source: Sci Rep. 2019 Jan 31;9:1103. doi: 10.1038/s41598-018-37387-9 (PMC6355868; doi:10.1038/s41598-018-37387-9)
Supplement: Supplementary file 1 — LaTeX Supplementary File [file 41598_2018_37387_MOESM1_ESM.zip › Fig/tumorExample.pdf]

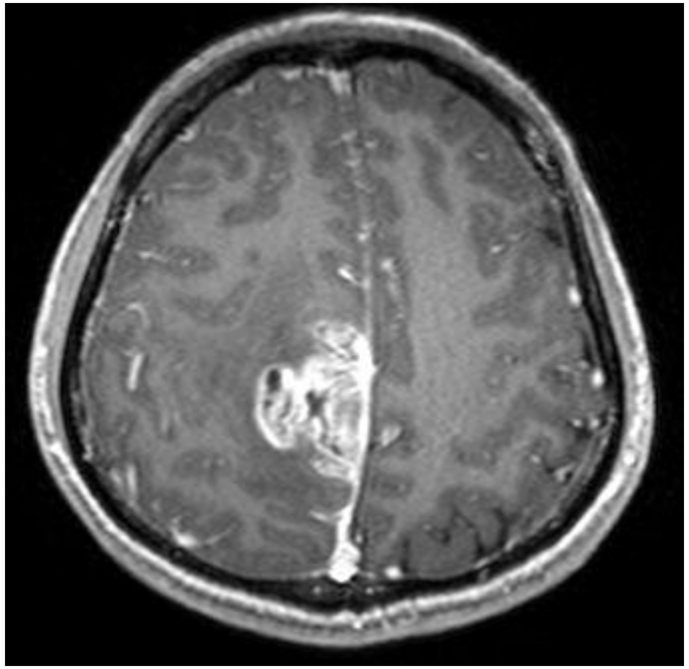
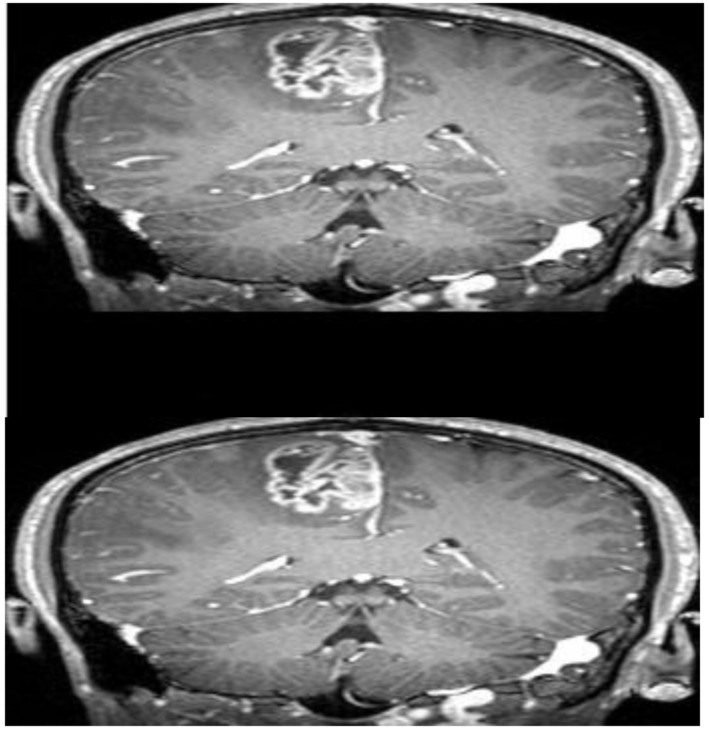

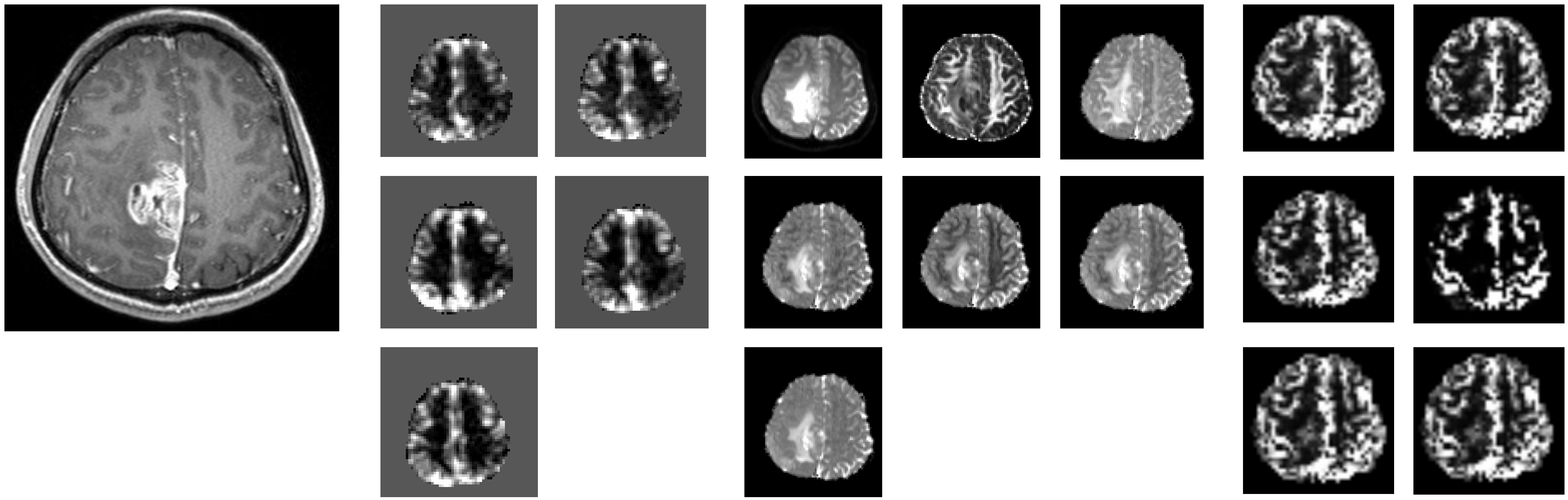

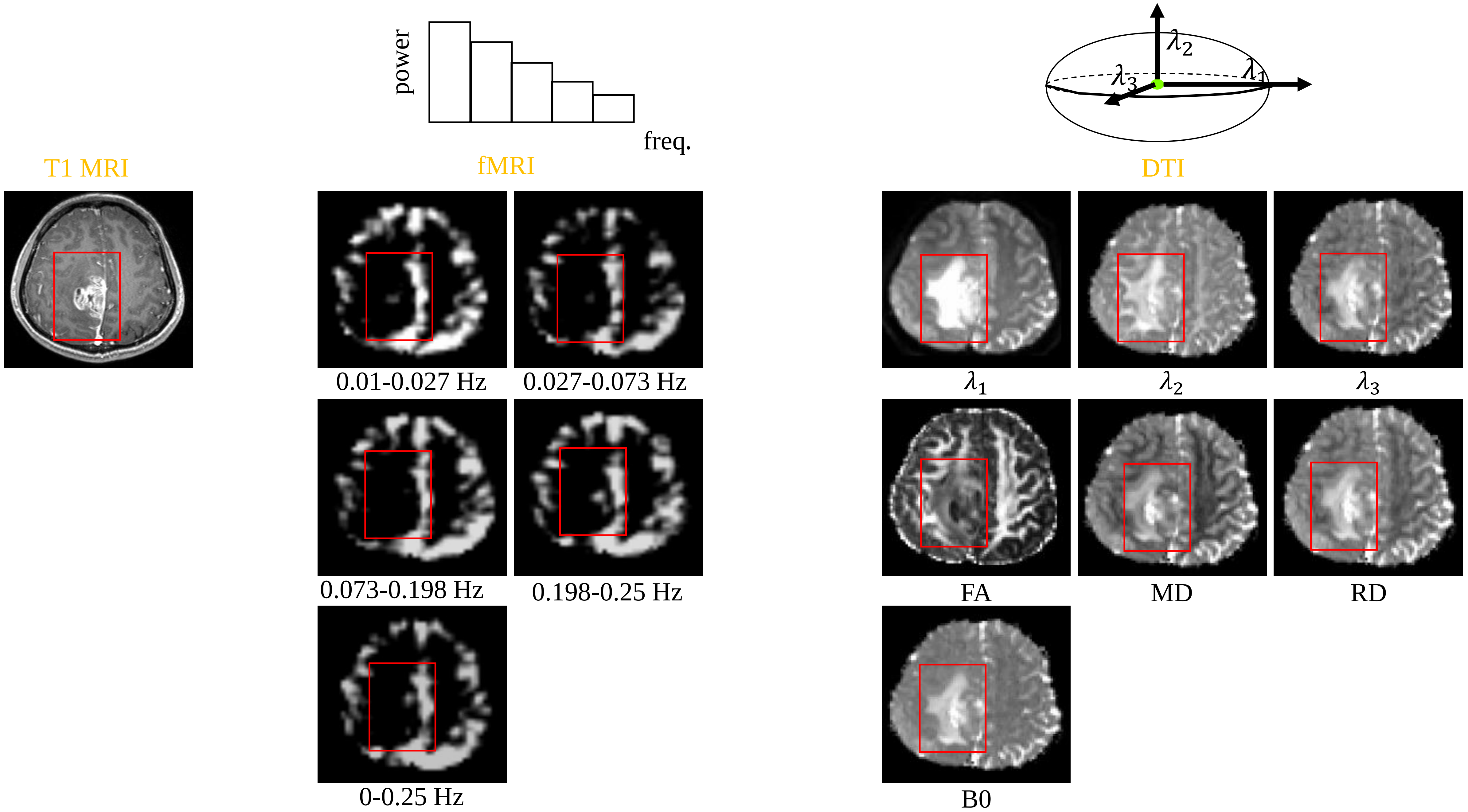
power
freq.
T1 MRI
fMRI
DTI
$\lambda_2$
$\lambda_3$
$\lambda_1$
0.01-0.027 Hz
0.027-0.073 Hz
0.073-0.198 Hz
0.198-0.25 Hz
0-0.25 Hz
$\lambda_1$
$\lambda_2$
$\lambda_3$
FA
MD
RD
B0

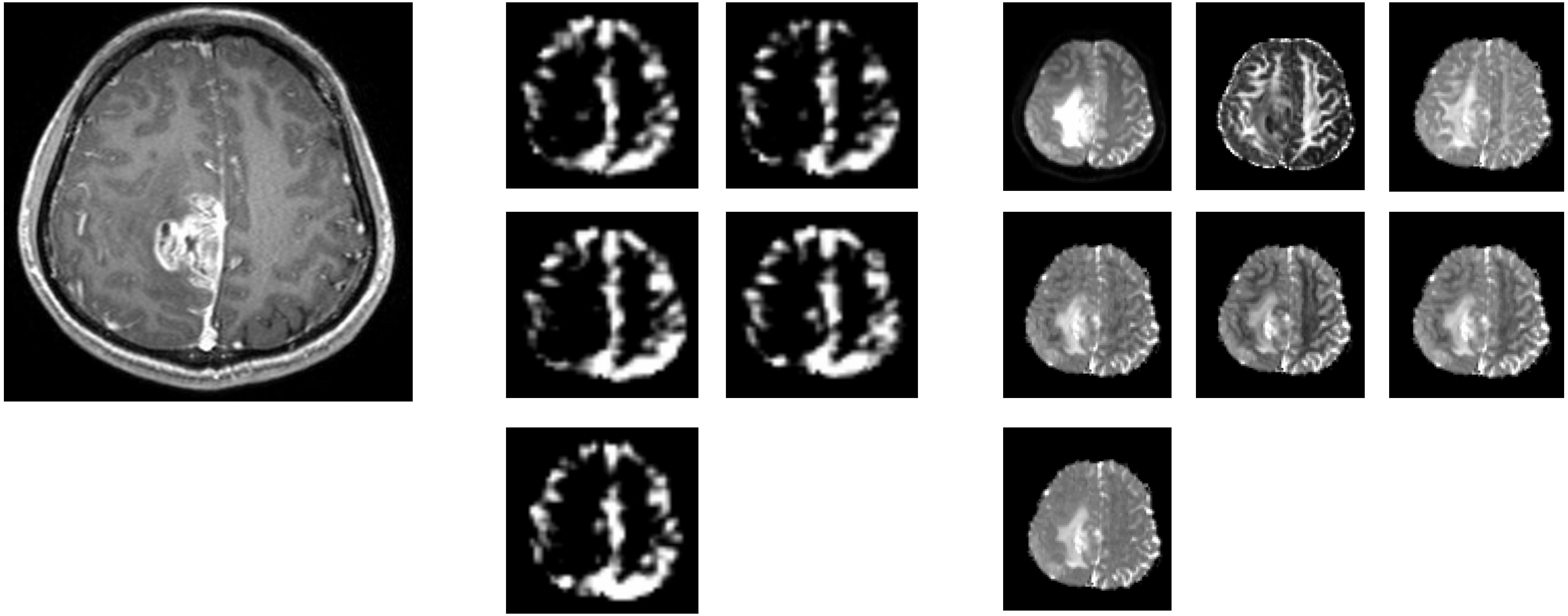

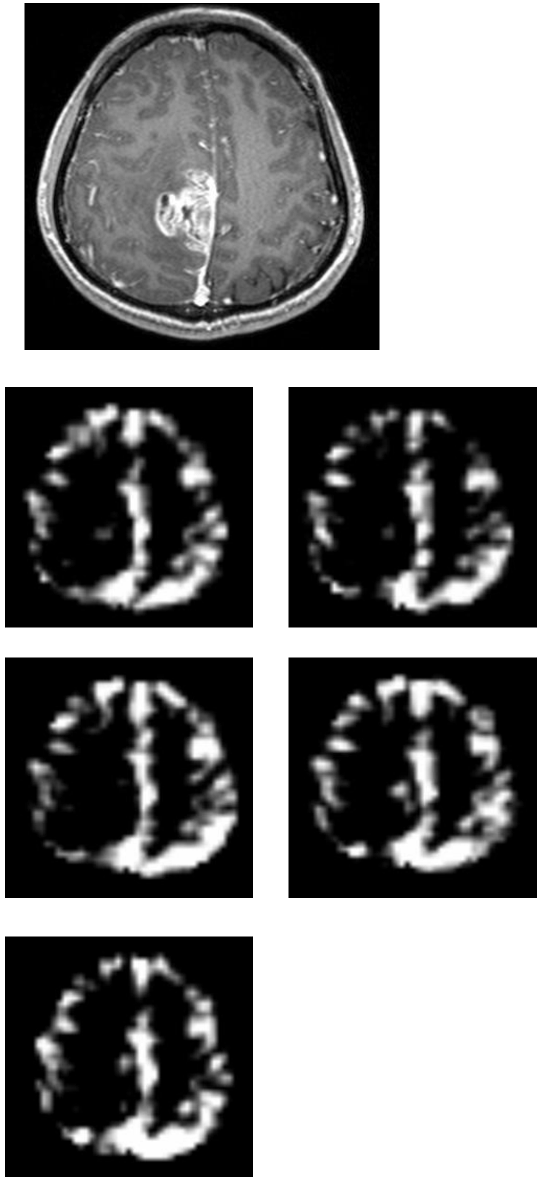

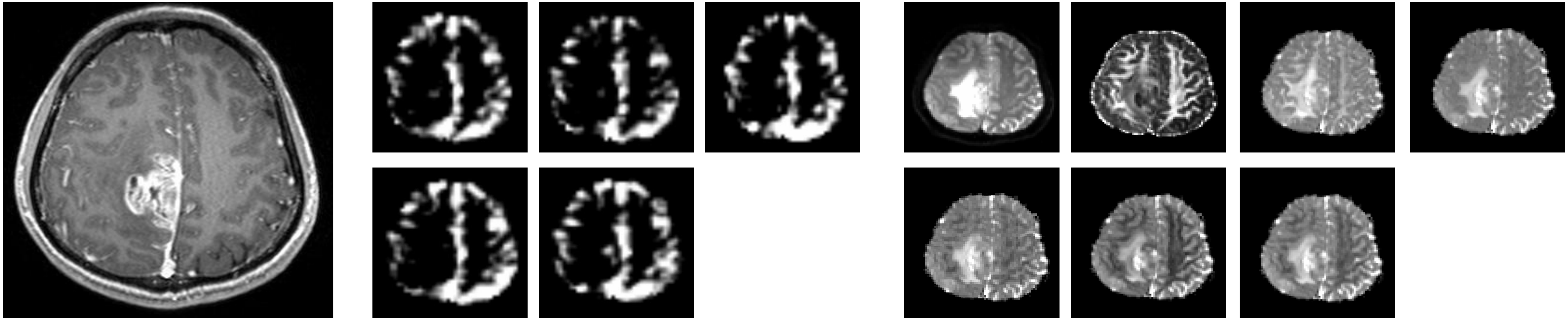

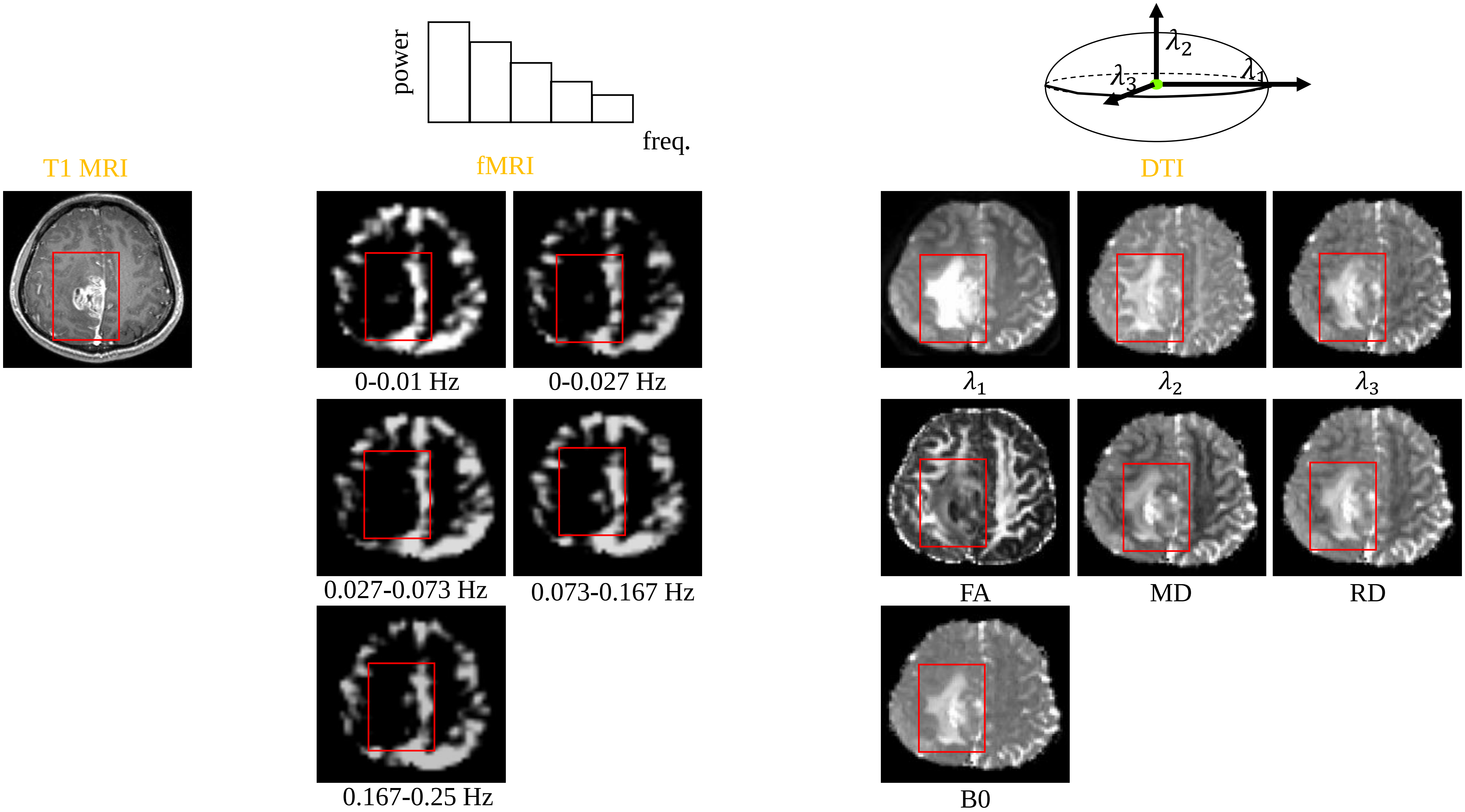
power
freq.
T1 MRI
fMRI
DTI
$\lambda_1$
$\lambda_2$
$\lambda_3$
0-0.01 Hz
0-0.027 Hz
0.027-0.073 Hz
0.073-0.167 Hz
0.167-0.25 Hz
$\lambda_1$
$\lambda_2$
$\lambda_3$
FA
MD
RD
B0

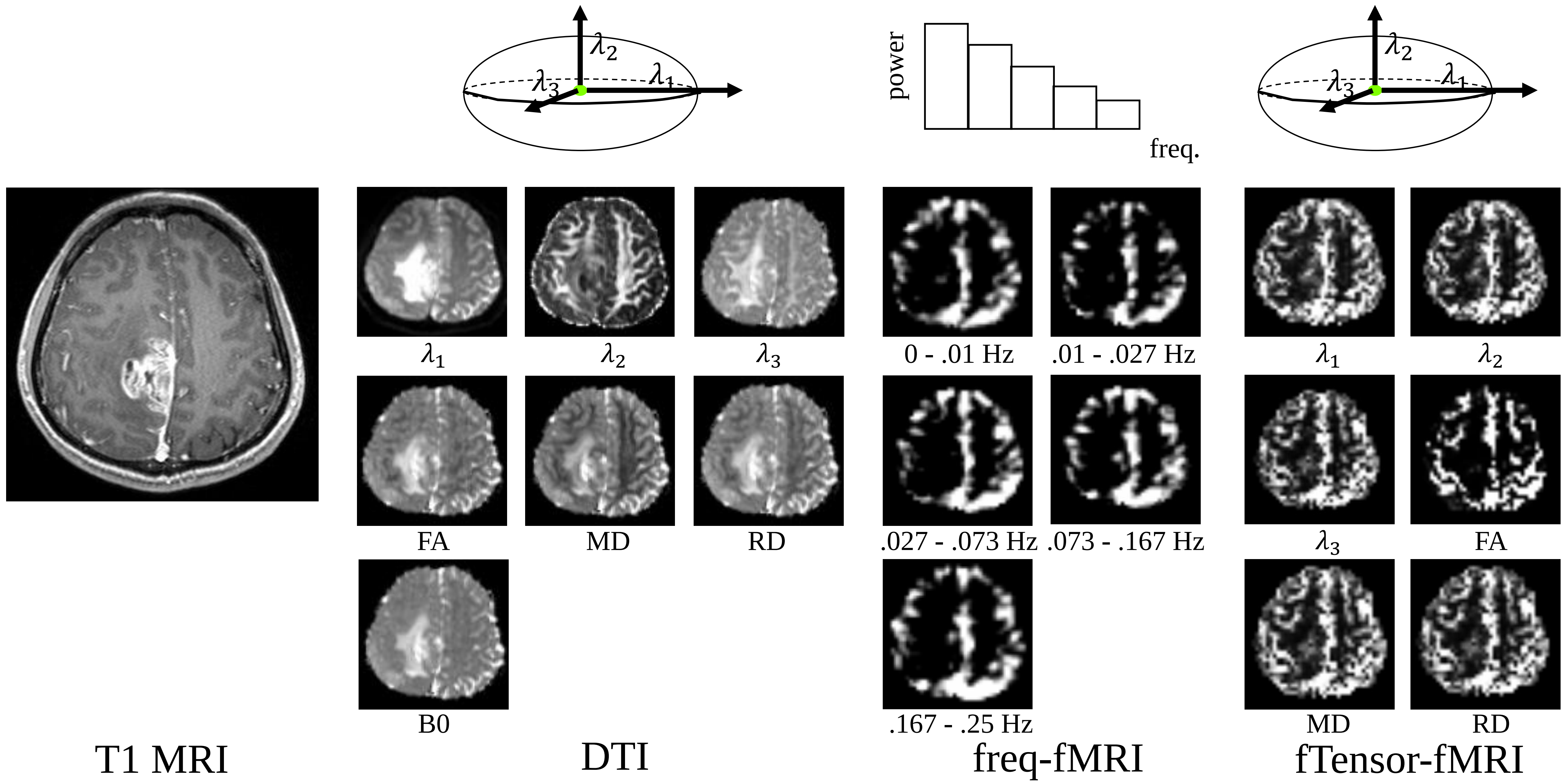

$\lambda_2$
$\lambda_3$
$\lambda_1$
power
freq.
$\lambda_2$
$\lambda_3$
$\lambda_1$
$\lambda_1$
$\lambda_2$
$\lambda_3$
FA
MD
RD
B0
0 - .01 Hz
.01 - .027 Hz
.027 - .073 Hz
.073 - .167 Hz
.167 - .25 Hz
$\lambda_1$
$\lambda_2$
$\lambda_3$
FA
MD
RD
T1 MRI
DTI
freq-fMRI
fTensor-fMRI

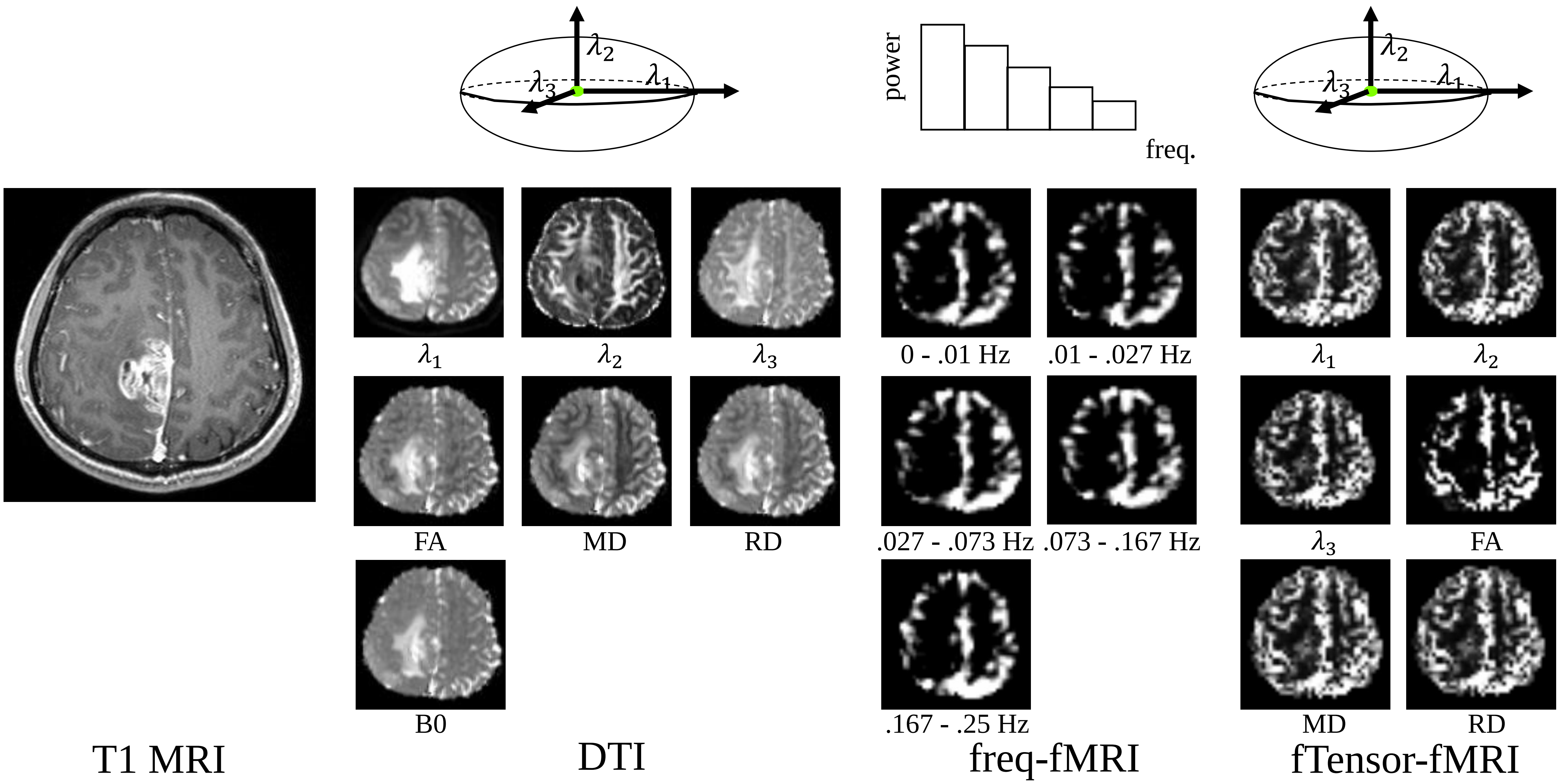

$\lambda_2$
$\lambda_3$
$\lambda_1$
power
freq.
$\lambda_1$
$\lambda_2$
$\lambda_3$
FA
MD
RD
B0
0 - .01 Hz
.01 - .027 Hz
.027 - .073 Hz
.073 - .167 Hz
.167 - .25 Hz
$\lambda_1$
$\lambda_2$
$\lambda_3$
FA
MD
RD
T1 MRI
DTI
freq-fMRI
fTensor-fMRI

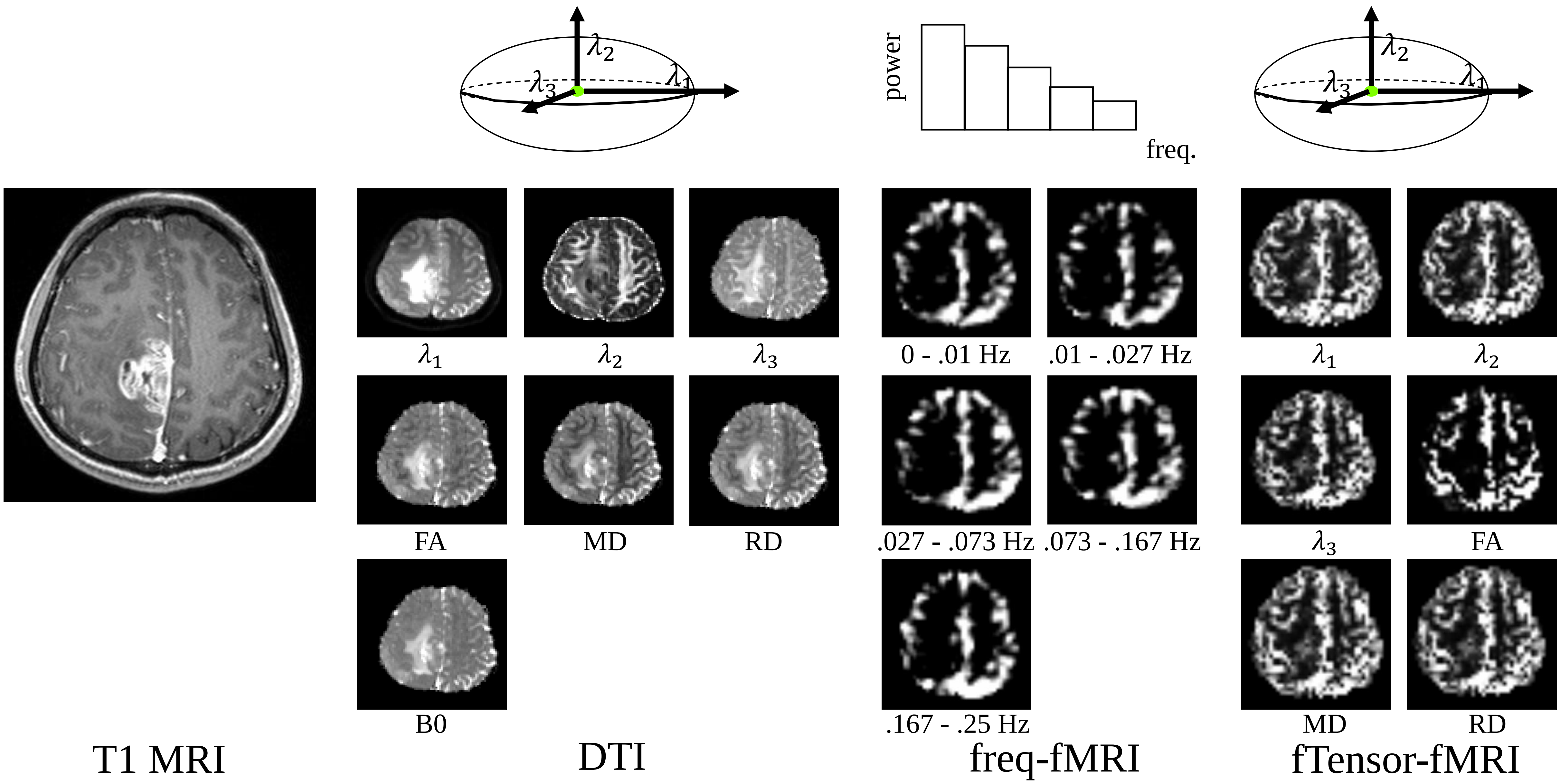

$\lambda_2$
$\lambda_3$
$\lambda_1$
power
freq.
$\lambda_2$
$\lambda_3$
$\lambda_1$
$\lambda_1$
$\lambda_2$
$\lambda_3$
0 - .01 Hz
.01 - .027 Hz
$\lambda_1$
$\lambda_2$
FA
MD
RD
.027 - .073 Hz
.073 - .167 Hz
$\lambda_3$
FA
B0
.167 - .25 Hz
MD
RD
T1 MRI
DTI
freq-fMRI
fTensor-fMRI

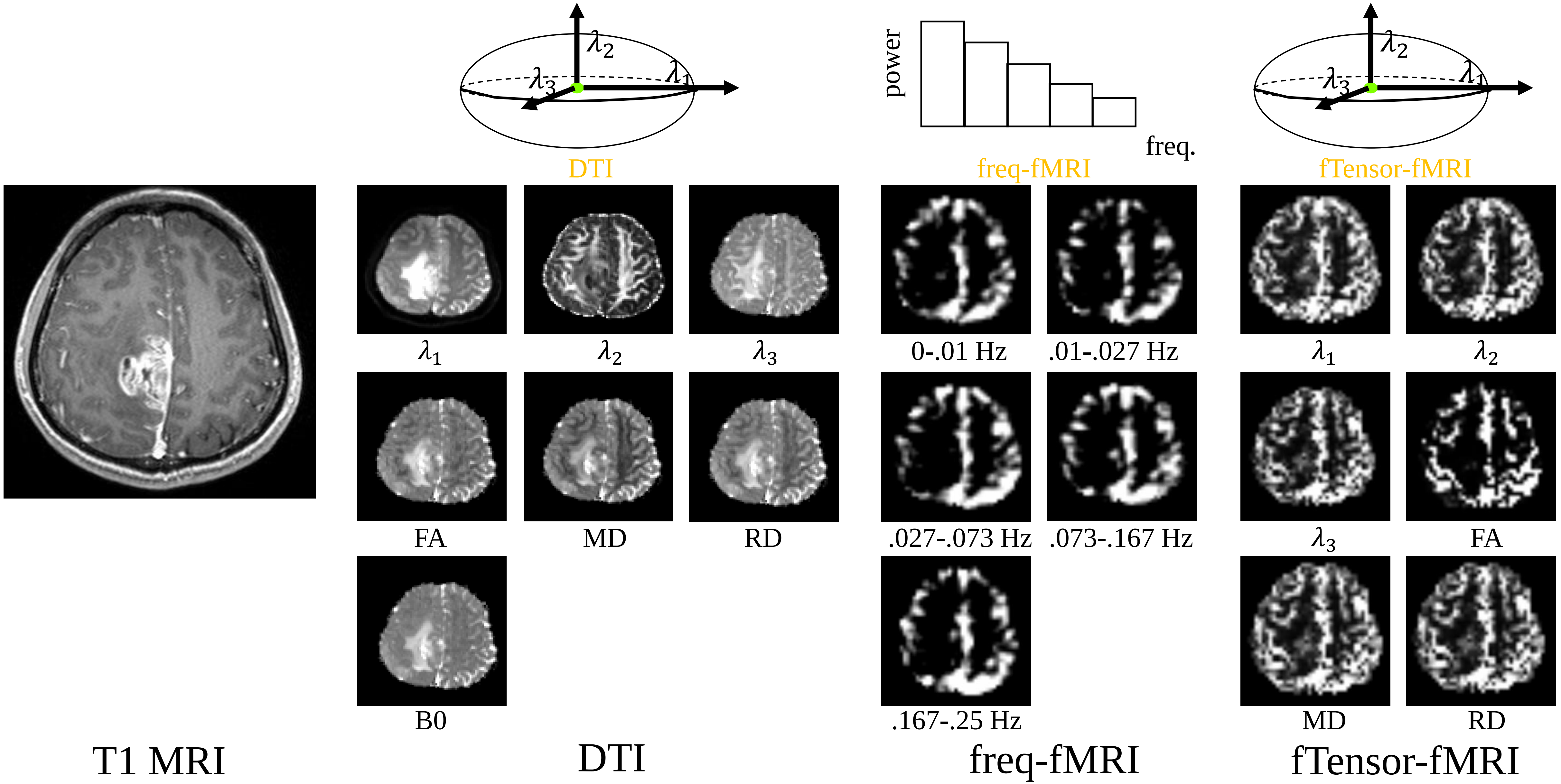

$\lambda_2$
$\lambda_3$
$\lambda_1$
power
freq.
DTI
freq-fMRI
fTensor-fMRI
$\lambda_1$
$\lambda_2$
$\lambda_3$
FA
MD
RD
B0
0-.01 Hz
.01-.027 Hz
.027-.073 Hz
.073-.167 Hz
.167-.25 Hz
$\lambda_1$
$\lambda_2$
$\lambda_3$
FA
MD
RD
T1 MRI
DTI
freq-fMRI
fTensor-fMRI
