# Supplementary material for: Multi-Channel 3D Deep Feature Learning for Survival Time Prediction of Brain Tumor Patients Using Multi-Modal Neuroimages
Source: Sci Rep. 2019 Jan 31;9:1103. doi: 10.1038/s41598-018-37387-9 (PMC6355868; doi:10.1038/s41598-018-37387-9)
Supplement: Supplementary file 1 — LaTeX Supplementary File [file 41598_2018_37387_MOESM1_ESM.zip › Fig/tumor_modality_new2.pdf]

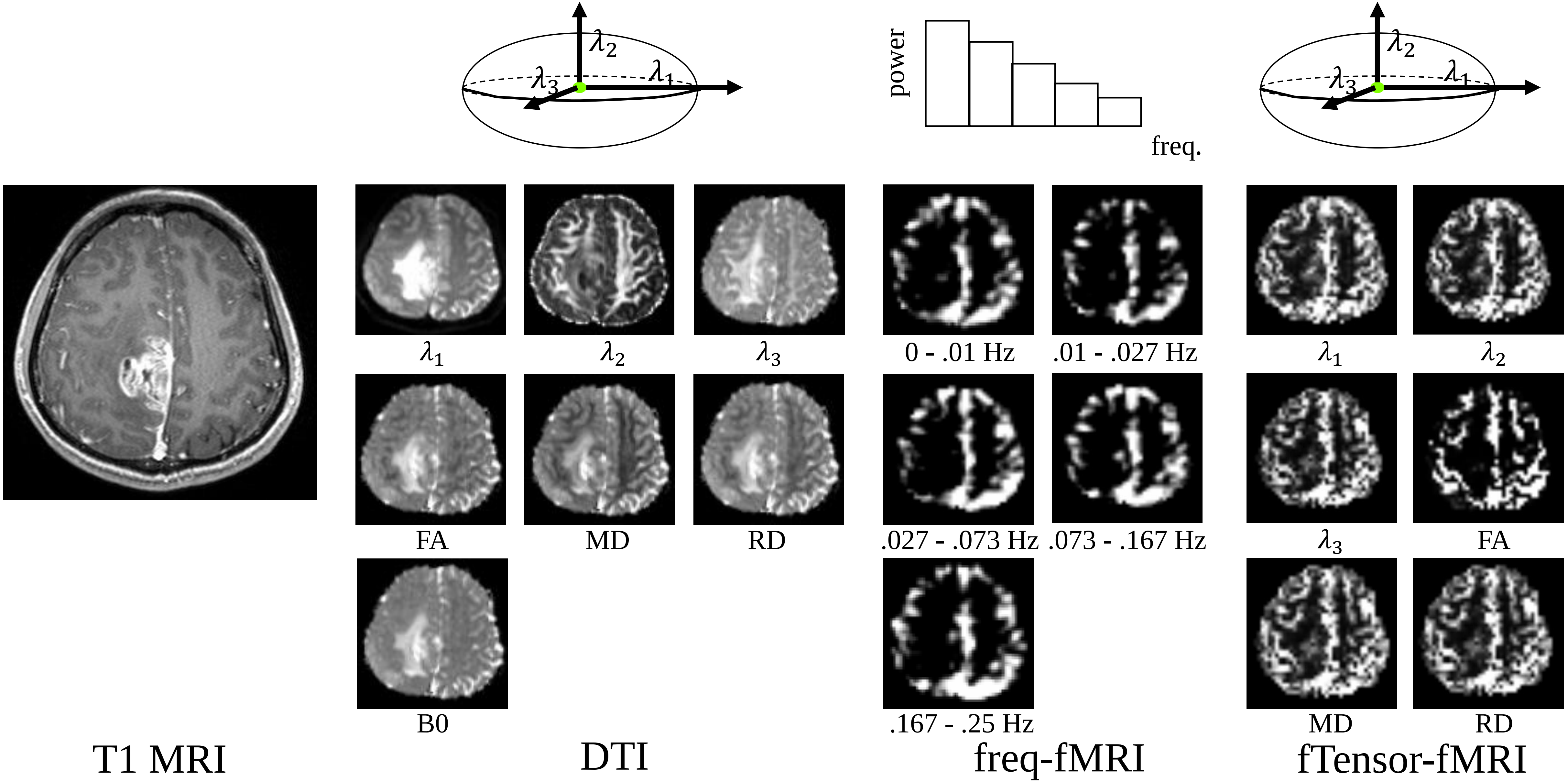

λ_2
λ_3
λ_1
power
freq.
λ_2
λ_3
λ_1
λ_1
λ_2
λ_3
FA
MD
RD
B0
0 - .01 Hz
.01 - .027 Hz
.027 - .073 Hz
.073 - .167 Hz
.167 - .25 Hz
λ_1
λ_2
λ_3
FA
MD
RD
T1 MRI
DTI
freq-fMRI
fTensor-fMRI
