# Supplementary material for: Multi-Channel 3D Deep Feature Learning for Survival Time Prediction of Brain Tumor Patients Using Multi-Modal Neuroimages
Source: Sci Rep. 2019 Jan 31;9:1103. doi: 10.1038/s41598-018-37387-9 (PMC6355868; doi:10.1038/s41598-018-37387-9)
Supplement: Supplementary file 1 — LaTeX Supplementary File [file 41598_2018_37387_MOESM1_ESM.zip › tumoros-scirep-majorrevision/tumoros-scirep-majorrevision.pdf]

Multi-Channel 3D Deep Feature Learning for Survival Time Prediction of Brain Tumor Patients Using Multi-Modal Neuroimages

Dong Nie[1,2,+], Junfeng Lu[3,4,+], Han Zhang[2,+], Ehsan Adeli[2], Jun Wang[2], Zhengda Yu[3,4], LuYan Liu[5], Qian Wang[5,*], Jinsong Wu[3,4,*], and Dinggang Shen[2,6,*]

[1]Department of Computer Science, University of North Carolina at Chapel Hill, NC 27514, USA
[2]Department of Radiology and BRIC, University of North Carolina at Chapel Hill, NC 27514, USA
[3]Department of Neurosurgery, Huashan Hospital, Fudan University, Shanghai 200040, China
[4]Shanghai Key Lab of Medical Image Computing and Computer Assisted Intervention, Shanghai 200040, China
[5]Med-X Research Institute, School of Biomedical Engineering, Shanghai Jiao Tong University, Shanghai, 200030, China
[6]Department of Brain and Cognitive Engineering, Korea University, Seoul 02841, Republic of Korea
[*]wang.qian@sjtu.edu.cn, wjsongc@126.com, dgshen@med.unc.edu
[+]these authors contributed equally to this work

ABSTRACT

High-grade gliomas are the most aggressive malignant brain tumors. Accurate pre-operative prognosis for this cohort can lead to better treatment planning. Conventional survival prediction based on clinical information is subjective and could be inaccurate. Recent radiomics studies have shown better prognosis by using carefully-engineered image features from magnetic resonance images (MRI). However, feature engineering is usually time consuming, laborious and subjective. Most importantly, the engineered features cannot effectively encode other predictive but implicit information provided by multi-modal neuroimages. We propose a two-stage learning-based method to predict the overall survival (OS) time of high-grade gliomas patient. At the first stage, we adopt deep learning, a recently dominant technique of artificial intelligence, to automatically extract implicit and high-level features from multi-modal, multi-channel preoperative MRI such that the features are competent of predicting survival time. Specifically, we utilize not only contrast-enhanced T1 MRI, but also diffusion tensor imaging (DTI) and resting-state functional MRI (rs-fMRI), for computing multiple metric maps (including various diffusivity metric maps derived from DTI, and also the frequency-specific brain fluctuation amplitude maps and local functional connectivity anisotropy-related metric maps derived from rs-fMRI) from 68 high-grade glioma patients with different survival time. We propose a multi-channel architecture of 3D convolutional neural networks (CNNs) for deep learning upon those metric maps, from which high-level predictive features are extracted for each individual patch of these maps. At the second stage, those deeply learned features along with the pivotal limited demographic and tumor-related features (such as age, tumor size and histological type) are fed into a support vector machine (SVM) to generate the final prediction result (i.e., long or short overall survival time). The experimental results demonstrate that this multi-model, multi-channel deep survival prediction framework achieves an accuracy of 90.66%, outperforming all the competing methods. This study indicates highly demanded effectiveness on prognosis of deep learning technique in neuro-oncological applications for better individualized treatment planning towards precision medicine.

Introduction

Brain tumors are one of the most lethal cancers. High-grade gliomas with World Health Organization (WHO) grades III and IV are the most deadly brain tumors with short overall survival (OS) time. Presurgical prognosis of the high-grade gliomas is highly desired in clinical practice for better treatment planning, but still challenging compared to low-grade gliomas (i.e., WHO grades I and II, for which generally long OS is expected). Presurgical OS prediction is traditionally believed to be affected by numerous factors, such as tumor location, histopathological types, patient's age, physical status, patient performance status and neurological disability[1–3]. Although generally corresponding to the short OS[4], recent molecular pathological studies have shown that the higher grade glioma patients with the same tumor histopathology may have significantly different OS[5]. These findings indicate that the traditional prognosis prediction based on the simple clinical and demographical information may not be adequately accurate[6–9]. Instead, based on the abundant non-invasive multi-modal neuroimaging data acquired prior to any invasive examination or surgery, a more accurate prognosis model for high-grade gliomas could be established, which is of great clinical importance and could benefit both treatment planning and patient care.

Recently, promising progress has been made using presurgical brain imaging and the radiomics features extracted from these images to study glioma prognosis, or to investigate phenotype-genotype association[10,11] for *indirect* prognostic studies. Among all presurgical neuroimaging modalities, T1-weighted magnetic resonance image (MRI) provides a 3D visualization of the brain structures with high soft-tissue contrast and high spatial resolution. Specifically, contrast-enhanced T1 MRI (where hyper-intensity suggests higher grade) has been widely used for imaging-based presurgical diagnosis and treatment planning. The rich appearance information depicted by this modality has also played an important role in prognostic studies[12–14]. For example, Pope et al. extracted 15 features from contrast-enhanced T1 MRI and found that the existence of non-enhancing regions indicated good OS while enhancing regions was not a valuable predictor[15]. Jain et al.[16] found that, using dynamic susceptibility contrast-enhanced T2*-weighted perfusion MR, increasing relative cerebral blood volume in non-enhancing regions could predict worsen OS. The same group further found that patients with high rCBV and wild-type EGFR mutation had poor overall survival[17]. However, Gutman et al. suggested that the volume of the enhancing lesions strongly indicated poor survival[12]. In a phenotype-genotype association study[18], more image features were found to be helpful for bifurcate survival curves, which included the volume of contrast-enhancing area again. In addition to the contrast-enhanced T1 MRI, diffusion tensor imaging (DTI) and functional MRI (fMRI) could also have prognostic values. DTI measures the anisotropic diffusivity of water molecules. It can be used to indicate edema and capture white matter microstructural alterations, which are helpful for OS evaluation. For instance, Saksena et al.found a potential relationship between various DTI metrics and survival time of glioblastoma patients[19]. Several survival studies have shown that DTI is statistically more effective to help separate glioblastoma patients into short and long survival groups than only using histopathologic information[20,21]. Although extensively used for presurgical functional mapping[22], fMRI has not been used for OS prediction yet. FMRI can measure brain function with blood oxygen level dependent (BOLD) signals and, similar to perfusion MRI, fMRI has also been used to characterize relative cerebral blood volume/flow[23]. Since highly malignant gliomas may have abnormal cerebrovascular reactivity[24], or altered regional blood flow due to neovascularization[25] and/or abnormal metabolism[26]. This imaging modality can also be used to predict OS.

The aforementioned imaging-based prognostic studies usually use handcrafted features that are carefully designed and manually extracted by well-trained and experienced clinicians (or automatically extracted using image processing techniques). Although such feature extraction is straightforward, the simplicity of such features prevents the rich information embedded in the multi-modal neuroimages from being fully utilized for OS prediction, because the handcrafted features are extracted based on previous studies or prior knowledge of diseases, and can also be limited to the existing image processing techniques. These feature descriptors could be biased and subjective to human interference. On the contrary, rich imaging phenotype information, which is beyond simple changes in image contrast/intensity, is deeply embedded and could be of essential prognostic value. The functional alternations within/around the visible lesion in MRI should also be extracted to further improve prognostic accuracy. Recently, rich radiomics features have further extended our knowledge on the roles of neuroimages in OS predictability[13]. However, the automatically extracted features in these studies are mostly based on the existing image processing algorithms or related with the lesions or their proximity[27]. There could be more sophisticated, high-level features that have better OS predictive values. In addition, the simple methodology in previous studies may also prevent the multi-modal images being well-integrated for OS prediction. Most existing works conduct univariate (i.e., independently considering each feature) or multivariate (i.e., jointly considering all features in a linear regression framework) analysis for prognosis[3,27]. The individual-level predictive capability (i.e., predicting OS for a single patient) of these models is limited by the group-level comparisons (e.g., identifying image features as biomarkers for either statistically partitioning patients into the long/short OS groups[3] or statistically better bifurcated of survival curves[13,14,28]). In clinical practice, however, survival time is expected to be individually predictable.

Similar to a radiologist who deliberates a prognostic suggestion after carefully review and comparison of all multi-modal images, we propose an objective and accurate computer-aided OS prediction framework for high-grade glioma patients. Our method is powered by popular and effective machine learning techniques, such that it is capable of extracting multi-modal and multi-channel neuroimaging features and effectively fusing them for individual OS prediction. We particularly use deep learning to extract features. With a convolutional neural network (CNN), a hierarchy of appearance features can be synthesized from low level to high level in a layer-by-layer manner[29,30]. Upon the convolutional parameters of the CNN are trained, with which the input raw image patches (i.e., small segments of whole-brain image) can be mapped to fit the target estimates (long/short OS). The mapping yields a highly sophisticated feature representation for the neuroimages, which is the key advantage of CNN compared to other machine learning methods. The CNN has shown superior performance on numerous visual object recognition and image classification studies[31]. It has also boosted the development of medical image analysis[32], including applications to tumor diagnosis[33]. In this paper, we propose a novel learning based method to predict OS of high-grade glioma patients: 1) We first automatically learn the high-level features for patches from multi-modal images (i.e., using the popularly acquired presurgical images, including contrast-enhanced T1 MRI, DTI and resting-state fMRI (rs-fMRI)) by training a supervised deep learning model at patch level; 2) We then train a binary support vector machine (SVM) model[34,35] based on the automatically

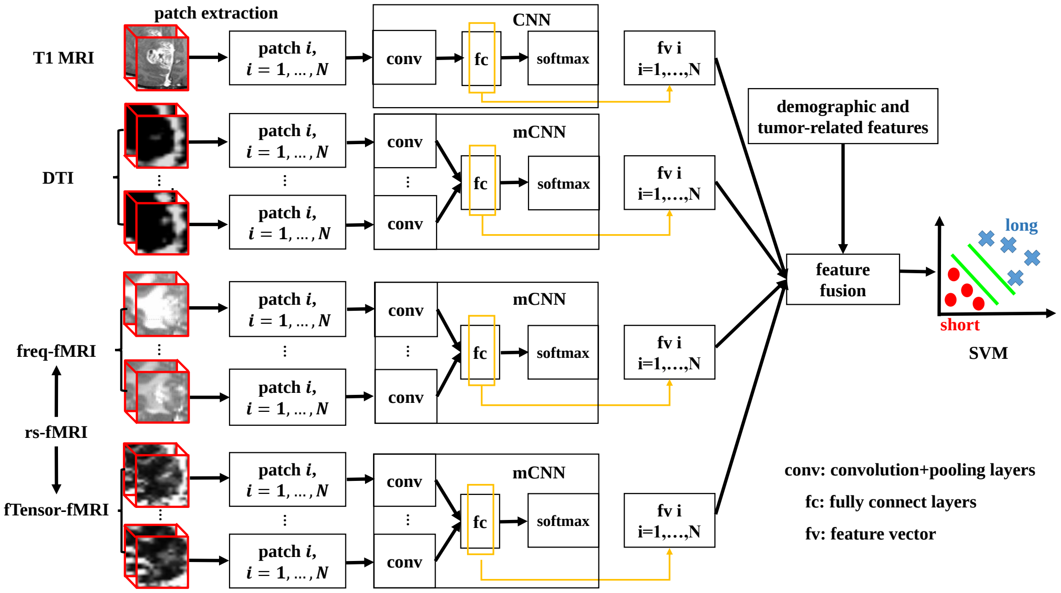

Figure 1. Schematic description of the proposed survival prediction framework for high-grade glioma patients, by using 1) 3D CNN-based deep learning to conduct feature learning and 2) an SVM for final prediction (long or short OS).

extracted semantic features (i.e., by concatenating patch-level features together to form patient-level features for each patient) to predict the OS for each patient. To well utilize neuroimage information, we calculate multiple diffusivity metric maps as multi-channel maps for DTI; also from rs-fMRI, we derive two types of multi-channel images using frequency information (freq-fMRI) of voxel-wise brain activity and inter-voxel local functional connectivity anisotropy (expressed as "functional tensor", or fTensor-fMRI), respectively. The flowchart of our OS prediction framework is illustrated by Fig. 1.

A preliminary version of this work has been presented at a conference[36]. Herein, we (i) extend our method by introducing an additional modality (fTensor-fMRI) to enhance the multi-modal multi-channel feature learning by providing more supplementary information, (ii) explore the impact of using the multi-modality information, (iii) investigate the impact of convolutional kernels: comparing 3D convolutional kernels with 2D convolutional kernels, (iv) compare the proposed supervised learned features with unsupervised extracted features on the classification task, and (vi) test on an extra 25-subject dataset.

Experiments and Results

Data Acquisition

Subjects: In this study, we included 68 patients with high-grade gliomas screened by presurgical imaging from the glioma image database (collected during 2010-2015) of Huashan hospital, Shanghai, China. We call this dataset training dataset. We also included another independent dataset with 25 patients (see **Validation on Independent Dataset**), which is the validation dataset to further validate our model. The inclusion criteria are listed in the following: 1) patients who have primary intracranial tumor but having not received any treatment before multi-modal MRI scan; 2) patients who have all three key imaging modalities (i.e., T1 MRI, rs-fMRI and DTI) with the same imaging parameters (see **Imaging Parameters**); and 3) with the screening thick-slice contrast-enhanced T1 MRI clearly showing the enhancing lesions (indicating high-grade gliomas). The exclusion criteria are patients 1) with any surgery, radiotherapy, or chemotherapy of brain tumor prior to image acquisitions; 2) with excessive head motion or presence of artifacts in any image. To avoid subjectivity, the T1 images were separately visually evaluated by three raters, only consensus result were used for decision making; 3) with irrelevant death causes (e.g., suicide) during follow-ups which may confound OS estimation; and 4) with inadequate follow-up period to determine the label of long or short OS. Specifically, OS is defined by the duration from the date when the patient received operation (i.e., the starting date of treatment) to the date of death (if applicable). The threshold is chosen to be 650 days and the patients are thus divided into two groups: short OS group and long OS group. This threshold is defined according to the median OS for the adult high-grade glioma patients[37]. The patients who were alive according to the latest follow-up but already lived longer than 650 days are also labeled as "long OS". Detailed patient information can be found in Table 1. Informed written consents were acquired from all the participants before imaging. The imaging study was also approved by the local ethical committee at Huashan hospital. The whole study was carried out in accordance with the approved guidelines. The images from a sample subject are shown in Fig. 2,

Table 1. Statistical information for the recruited patients.

Variables	Value	Variables	Value
**Age (years)**		**WHO, histological type (%)**	
Mean±std	51.4±14.5	III, anaplastic astrocytomas	17 (25)
Range	16-74	III, anaplastic oligodendrogliomas	9 (13.2)
**Males/females (%)**	48/68(70.6)	III, anaplastic ependymomas	1 (1.5)
**Hemisphere (%)**		IV, glioblastoma	41 (60.3)
Left	50 (73.5)	**Tumor size** (mm^3)	
Right	16 (23.5)	Mean±std	53.9±40.1
Bilateral	2 (3)	Range	1.7 200
**Main location (%)**		**Preoperative epilepsy (%)**	27/68(39.7)
Occipital	4 (5.9)	**Overall survival time(%)**	
Temporal	20 (29.4)	$\leq$ 650 days	29 (42.6)
Parietal	7 (10.3)	$\geq$ 650 days (dead)	27 (39.7)
Frontal	31 (45.6)	$\geq$ 650 days (live)	12 (17.6)
Insula	6 (8.8)		

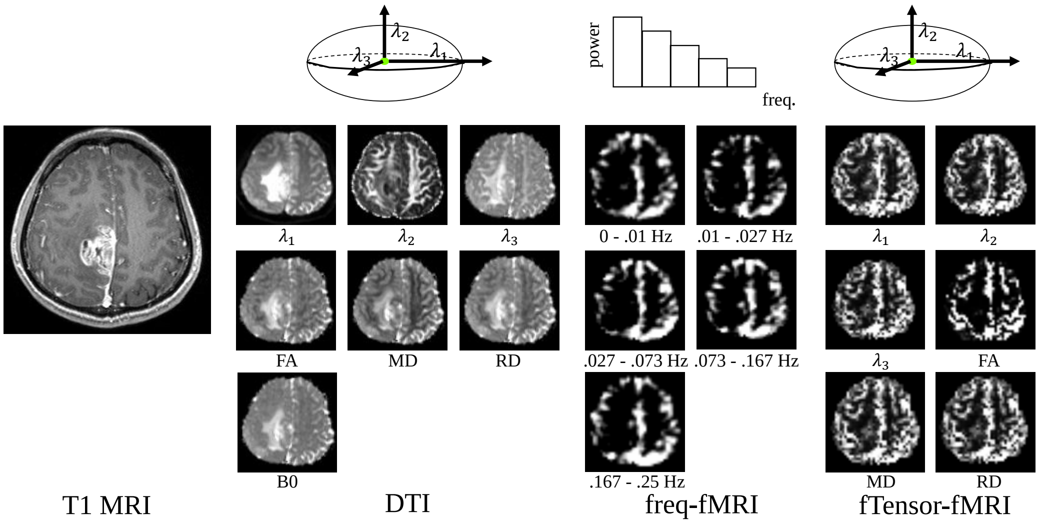

Figure 2. A sample glioblastoma patient in our dataset. T1 MRI is presented as a single metric map, while DTI and rs-fMRI are presented as multi-channel metric maps.

from which we can see the contrast-enhanced T1 MRI (presented in single channel), and the multi-channel metric maps derived from DTI and rs-fMRI. The detailed multi-channel metric map calculation will be described later.

Imaging parameters: All the multi-modal images are acquired by a 3T MRI scanner (MAGNETOM Verio, Siemens Healthcare, Siemens AG, Germany) at Huashan hospital. The image data collected from each patient subject include T1 MRI (TR, 1900 ms; TE, 2.93 ms; flip angle, 9°; FOV, 250×250 mm^2; matrix size, 256×215; slice thickness, 1 mm; acquisition average, 1), DTI (TR, 7600 ms; TE, 91 ms; slice thickness, 3 mm; inter-slice space, 0 mm; b-value, 1000 s/mm^2; NEX, 2; FOV, 230×230 mm^2; matrix size, 128×128; voxel size, $1.8 \times 1.8 \times 3$ mm^3; number of gradient directions, 20), and rs-fMRI (TR, 2000 ms; TE, 35 ms; flip angle, 90°; number of acquisitions, 240 (8 min); slice number, 33; slice thickness, 4 mm; inter-slice gap, 0 mm; FOV, 210×210 mm^2; matrix size, 64×64; voxel size, $3.4 \times 3.4 \times 4$ mm^3).

Treatment: All patients have been treated according to the clinical guideline for adult high-grade gliomas. All cases have achieved the maximal safe tumor resection by the same neurosurgeon (JW, with 20+ years' experience) with intraoperative neurophysiological monitoring, which ensures the consistency of the surgical treatments across subjects. All tumors have been totally or gross-totally removed according to postsurgical imaging. In our study, the patients received concurrent high conformal radiation therapy and chemotherapy with Temozolomide followed by six cycles of Temozolomide according to Stupp's regimen[38]. For radiotherapy, each patient received fractionated focal irradiation in daily factions of 2 Gy given 5 days per week for 6 weeks, for a total of 60 Gy.

There is no significant difference between two OS groups in preoperative tumor volumes ($p = 0.55$) and extension of resection ($p = 0.22$). There is also no significant difference between two OS groups in the postoperative treatment ($p = 0.82$). Of note, this paper aims to develop a novel method that can preoperatively predict long/short survival outcome. We will show that, given the guideline and suggested following treatment, one can potentially predict the OS from the presurgical imaging data. Accordingly, the prediction of OS can be made for the presurgical planning, as the decision is made prior to the surgery. We by no means advocate that treatment is irrelevant to the final outcome.

Data Preprocessing

We preprocess the images for convincing quantitative study. Of note, the three modalities of images are spatially co-registered (specifically, we register all other image modalities to T1-weighted image for each subject), but not further registered to the Montreal Neurological Institute (MNI) standard space, to avoid artifacts caused by nonlinear deformation (see details below).
T1 MRI: For each subject, the tumor as well as its close surrounding area is extracted by a rectangular bounding box. The bounding box is manually drawn, and further verified by a second radiologist to assure that all tumor lesions are included. Since both rs-fMRI and DTI images are co-registered to the T1 MRI of the same subject, the bounding boxes can be applied to other multi-channel metric maps of the same subject.
DTI: We use PANDA[39] to process DTI data. The main procedures include brain extraction, eddy current correction, and diffusion tensor and diffusivity metric calculation. In particular, we compute 6 diffusivity metric maps: fractional anisotropy (FA), mean diffusivity (MD), the first/second/third eigenvalue of the tensor (λ_1, λ_2, λ_3), and radial diffusivity (RD) (with more details in this paper[40]). Together with B0 (b = 0 s/mm^2) map, the 7 metric maps constitute 7 channels, which are co-registered to T1 MRI per subject.
rs-fMRI: Data preprocessing is performed by DPARSF[41], including removal of the first 5 volumes, slice timing, head motion correction, spatial smoothing, linear trend removal, and regressing out the nuisance covariates that consist of averaged white-matter and cerebrospinal-fluid signals. Frequency-specific BOLD fluctuation power maps are calculated in five non-overlapping frequency bands[42,43] (see Fig. 2), resulting in 5 metric maps (freq-fMRI). Note that these 5 maps mainly focus on grey matter. To also extract functional features in white matter, we propose to use the functional connectivity tensor (fTensor-fMRI)[11,44] to provide functional information in white matter. The fTensor-fMRI was originally proposed in the work[44] to measure the structured spatiotemporal relationship among the BOLD signals of neighboring voxels in white matter, which shows the anisotropic pattern that is generally consistent with the diffusion anisotropy derived from DTI in major fiber bundles. The fTensor-fMRI has demonstrated to be able to detect functional changes in white matter, caused by task stimulation[44] and anesthesia[45]. Since gliomas grow in white matter, which could alter the fTensor-fMRI, we decide to adopt this metric (mainly focusing on the white matter) together with freq-fMRI (mainly focusing on the grey matter) to jointly predict OS. Like the metric maps derived from diffusion tensor for DTI, we also compute λ_1, λ_2, λ_3, FA, MD and RD maps based on fTensor for rs-fMRI, which results in 6 additional metric maps (thus totally 11 metric maps for rs-fMRI). Of note, it is the first time that fTensor-fMRI has been used in clinical applications.

For all channels of the metric maps from the three imaging modalities, the bounding box is applied. Then, the intensities inside the bounding box are normalized per metric map. The 3D metric maps within the bounding box are rescaled to the same dimension ($64 \times 64 \times 64$) to facilitate the subsequent deep learning.

Experimental Settings

As described earlier, we use our 3D CNN architectures, which is implemented by the widely used deep learning framework Caffe[46], to extract features from multi-modal brain images and their multi-channel metric maps in a supervised manner. These features are expected to classify individual image patches according to the survival of the patient. Then, the high-level features of all patches of the patient, as well as the important limited demographic and tumor-related features, are integrated to train the SVM classifier for the survival time prediction of the patient. As mentioned in the **Method** section, the patch-based features are processed through Principal Component Analysis (PCA) for feature reduction and Sparse Representation (SR)[47] for feature selection. Specifically, the number of the features from fc6 for each metric map is $8 \times 256 = 2048$ (note that we extract 8 non-overlapping patches uniformly within the bounding box of each metric map for a subject), and the number for fc7 is $8 \times 2 = 16$. With PCA, we preserve the principal components up to 99% and reduce the feature numbers of fc6 for the T1 MRI, DTI, freq-fMRI and fTensor-fMRI to 9, 14, 12, 16, respectively, as well as the feature numbers of fc7 to 5, 7, 7, 8, respectively. With SR, we set the balance parameter to 0.2 with the SLEP package[48]. Usually, there are 3, 10, 8, 7 selected features from fc6 for the T1 MRI, DTI, freq-fMRI and fTensor-fMRI, respectively; as for fc7, the corresponding numbers are 3, 5, 4, 4, respectively. For the SVM[35], we chose the L1-regularized logistic regression and set the cost parameter to be 1.

Experimental Results

Cross-Validation Experiments: We use 10-fold and 3-fold cross-validation upon 68 patient subjects. That is, for each testing fold, the remaining other folds are used to train both the single-channel CNN (for T1 MRI) and mCNN (for DTI and fMRI), as

Table 2. Performance evaluation of different features and selection/reduction methods with 3-fold and 10-fold cross validation, respectively. * Significantly improved ($p < 0.05$) performance compared with "dtf" only method, as measured by McNemar's test.

Method	**ACC** (%)	**SEN** (%)	**SPE** (%)	**PPR** (%)	**NPR** (%)	Cross Validation
dtf	62.96	66.39	58.53	63.18	65.28	
fc7*	81.04	85.87	77.94	73.28	89.42	
fc6-PCA*	80.78	84.88	77.04	76.03	85.37	
fc6-SR	77.11	85.39	71.01	67.25	86.53	3-fold
dtf + fc7*	**90.66**	96.77	**85.04**	**85.82**	96.31	
dtf + fc6-PCA*	**90.27**	**96.48**	84.76	**84.98**	94.05	
dtf + fc6-SR*	86.68	93.51	82.39	78.64	**95.43**	
dtf	63.23	67.24	60.25	60.71	66.21	
fc7*	82.35	86.20	79.48	75.76	88.58	
fc6-PCA*	80.88	85.27	76.92	74.52	86.26	
fc6-SR	77.94	85.60	71.80	69.35	87.04	10-fold
dtf + fc7*	**90.46**	**95.82**	85.78	**84.81**	95.59	
dtf + fc6-PCA*	**90.17**	94.83	**85.90**	84.33	**95.71**	
dtf + fc6-SR*	86.76	93.10	82.05	79.41	94.12	

well as the SVM. The performance measures averaged over all the folds are reported in Table 2, including accuracy (ACC), sensitivity (SEN), specificity (SPE), positive predictive rate (PPR), and negative predictive rate (NPR).

Incorporating both deeply learned (with the proposed CNNs) and limited demographic & tumor-related features (dtf) leads to the best classification accuracy of 90.46%. In contrast, using dtf alone, we obtain just an accuracy of 63.23%. Regarding the sensitivity and the specificity, we know that the higher the sensitivity, the lower the chance of misclassifying the short survival patients; on the other hand, the higher the specificity, the lower the chance of misclassifying the long survival patients. The proposed feature extraction method resulted in an approximately 30% higher sensitivity and specificity, compared to the limited demographic and tumor-related features. Interestingly, our model predicts the short survival patients with more confidence than the long survival patients.

To further investigate the effectiveness of our proposed method, we also draw a Kaplan-Meier plot based on the model output (i.e., the hard classification labels) in Fig. 3. We can see that the survival curves of the two groups are well separated ($p < 0.0001$, log-rank test). The result indicates that our model can well separate subjects with long OS from those with short OS. It should be noted that the K-M plot we plotted is not based on the two values of a single explicit variable nor those of certain combined explicit variables, but the weighted combination of the deep learning features and the subsequent "hard" classification result. Therefore, our K-M plot should be interpreted carefully.

Besides the deep learned features and the limited demographic & tumor-related features, we also extract the radiomics features using traditional unsupervised feature learning methods, such as Haar features and SIFT features. We have reported the details in Section "Comparison with Unsupervised Feature Extraction Approaches". In addition, we also compare CNN alone, CNN + SVM (our method) and SVM alone (with the above radiomics features), and you can refer to "Impact of Combining CNN and SVM" for details.

Validation on Independent Dataset: To further validate the effectiveness of our proposed algorithm on predicting OS for gliomas patients, we test our trained model on a newly collected dataset. This newly collected dataset consists of 25 patients; each of them has the same modalities (channels) with the dataset described in Section **Data Acquisition**. The statistical information about the 25 patients are shown in Table 3. We preprocessed the data by following the same procedures as described in Section **Data Preprocessing**.

With preprocessed data, we adopt the trained neural networks to extract feature representation for all these 25 patients. Then, we apply the trained SVM model for classification based on the extracted features (Note that we only consider limited demographic and tumor-related features and fc7 features as the final features for the SVM model). The experimental results on such an independent dataset are presented in Table 4.

The performances reported in Table 4 are generally consistent with those in Table 2, especially for the accuracy, sensitivity and specificity. This further proves the robustness of the proposed method.

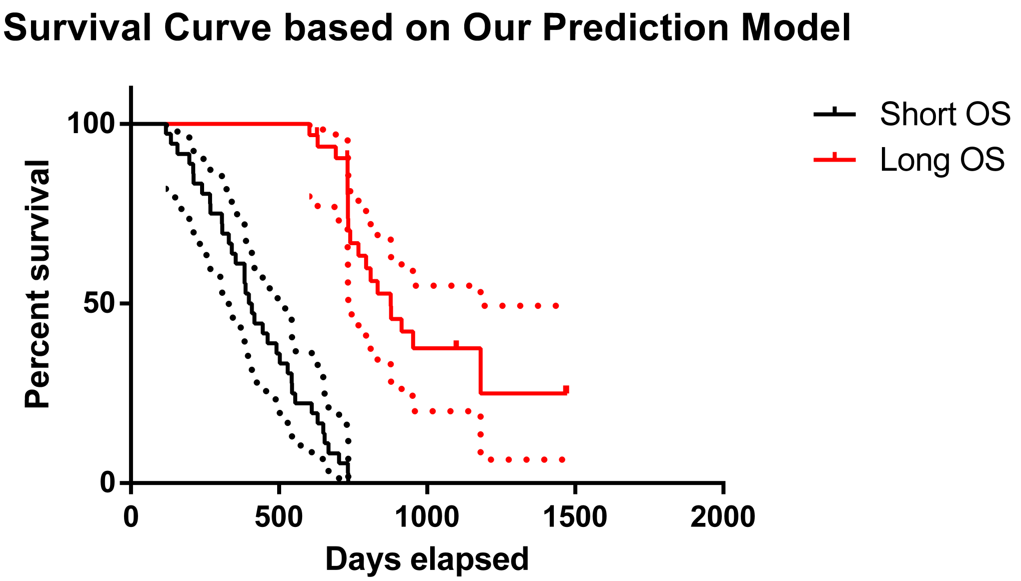

Figure 3. Kaplan-Meier plot of the two groups' survival data based on our predicted results. Dotted line indicates the 95% confidence interval.

Table 3. Statistical information for the newly recruited 25 patients (validation dataset).

Variables	Value	Variables	Value
**Age (years)**		**WHO, histological type (%)**	
Mean±std	50.2±13.0	III, anaplastic astrocytomas	6 (24)
Range	23-68	III, anaplastic oligodendrogliomas	3 (12)
**Males/females (%)**	16/9	III, anaplastic ependymomas	1 (4)
**Hemisphere (%)**		IV, glioblastoma	15 (60)
Left	16 (64)	**Tumor size** (mm^3)	
Right	8 (32)	Mean±std	35.8±32.3
Bilateral	1 (4)	Range	8.4 99.3
**Main location (%)**		**Preoperative epilepsy(%)**	27(39.7)
Occipital	1 (4)	**Overall survival time(%)**	
Temporal	6 (24)	≤650 days	9 (36)
Parietal	2 (8)	≥650 days (dead)	3 (12)
Frontal	14 (56)	≥650 days (live)	13 (52)
Insula	2 (8)		

Table 4. Performance comparison across different features for the newly collected 25 patients. * Significantly improved ($p < 0.05$) performance compared with "dtf" only method, as measured by McNemar's test.

	**ACC** (%)	**SEN** (%)	**SPE** (%)	**PPR** (%)	**NPR** (%)
dtf	68	66.67	68.75	54.55	78.57
fc7*	80	77.78	81.25	75	86.67
dtf + fc7*	**88**	**88.9**	**87.5**	**80**	**93.3**

Discussion

General Discussion

Accurate pre-operative prognosis for this high-grade glioma can lead to better treatment planning. Conventional survival prediction based on clinical information is prone to be subjective and sometimes could be not accurate enough. In this paper, we propose a multi-modality multi-channel deep learning method to automatically learn feature representations for the imaging

data and then a binary SVM model for the final tumor OS classification. Due to the use of powerful deep learning model, we can learn useful features from imaging data automatically. It can thus avoid being subjective if self-designing the features by radiologists, and will be able to explore some useful but hard-to-design features. Furthermore, our proposed deep feature learning method can be adapted to both single-channel and multi-channel imaging data. This is a huge advantage in clinical application as it is common that medical imaging data has uncertain number of channels. However, we by no means aim to underrate and criticize the traditional OS prediction model, but to test the feasibility of deep-learning-based OS prediction model as this type of methods has many advantages such as automatic feature learning, high-level feature learning, better ability to fuse multi-channel images, and so on.

Other studies also used conventional features, or radiomics features, but may include more features, such as KPS (daily living score), resection percentage, and some genomics features (e.g., IDH1 or MGMT). But obtaining these features will involve enormous work and resources. We would like to provide the reasons why we did not include them as below. First, KPS score is based on patients' self-report, which could be less objective. Moreover, all our patients have KPS scores larger than 90, making this factor count for very little variability of the survival data. Second, resection percentage is a treatment-related factor, which is beyond the scope of this study (i.e., to predict survival time based on presurgical data). Moreover, as described in Sec. "Treatment", all tumors have been totally or gross-totally removed, indicating that the optimized treatment has been achieved for all the patients. Therefore, our goal can be simply summarized as: to predict OS based on deep learning upon tumor multimodal imaging obtained presurgically, given optimized treatment following the guideline later on. Third, the genomic data is not available for most of the subjects, given the commencement of the study is early (i.e., since year 2010). Collectively, to achieve our preset research goal, and to simply convey our proposed method, i.e., to demonstrate the feasibility of DL-based feature selection for prognosis, we would rather prefer the current comparison strategy.

More importantly, our proposed framework is able to work well on a small dataset. In our study, the minimum unit or a sample is a patch in the feature learning stage, rather than a whole brain image. That is, for each subject, we can extract hundreds of patches (with the same label); therefore, we can eventually have enough samples (i.e., patches) to train the neural networks. In other words, we train the networks at the patch level, rather than a whole-image (or subject) level. After training the neural networks, we then train a SVM with the learned "deep features" (as they have been learned from deep convolutional neural networks) to classify the patients with short or long overall survival (OS) time. Because the features learned from the deep learning framework are more accurate and also at much higher level, the following SVM could have better performance than the SVMs using features extracted by traditional methods.

Moreover, our proposed framework can fuse multi-modality information so that it can fuse more information from different imaging modalities to determine the final classification. The results from additional experiments on this issue are detailed in the **Experiments and Results** section.

Contribution of Multi-Modality Information to OS Prediction

We run the same proposed framework for extracting features from each single modality, and train SVM using the extracted features. In this way, we can justify the importance of fusing multi-modal imaging data in predicting OS. The quantitative results are shown in Fig. 4. Among the single modality classification performances, the features from rs-fMRI yield the best performance among all single modalities, i.e., about 82.63% of accuracy (with significant improvement compared with the "dtf" only method, $p < 0.05$, McNemar's test). However, as it is obvious from the results, we can benefit about 7% improvement of accuracy by fusing multi-modal images, when using our proposed framework.

We also conduct experiments by comparing the outcome prediction performance using 3D CNN in our model against the case of using traditional 2D CNN. The result shows significant advantage of using 3D CNN (with the details given in Section **Comparison with 2D CNN based Approaches**). Another experiment shows the comparison result between supervised feature extraction method (in our proposed method) and traditional unsupervised feature extraction approaches (i.e., adopted in Radiomics studies), indicating the superiority of supervised feature extraction to unsupervised feature extraction (see details in the Section **Comparison with Unsupervised Feature Extraction Approaches**).

The Role of the Features from Each Modality

To analyze the importance of the features for predicting OS, we also calculate the number of the features selected from each modality in the prediction based on multi-modal images. To do this, we use the L1-regularized SVM for classification, for internally enforcing the selection of the most discriminative features from the outputs of the fc7 layers of the CNNs. The average numbers of the discriminative features selected for T1 MRI, DTI, freq-fMRI and fTensor-fMRI are 1, 4, 4, and 4, respectively. As can be seen, the fMRI and DTI data contribute more significantly in building a better prediction model, compared to the T1 MRI. However, for the T1 MRI, we only have a single channel of data, while multiple channels for the other modalities. Therefore, we further normalize these numbers by the total number of the channels from the corresponding modality. The normalized measures are 1, 0.57, 0.8 and 0.67, respectively, for the four (sub-)modalities. In this sense, the results then show that T1 MRI encodes relatively more information for OS prediction, compared to DTI and fMRI.

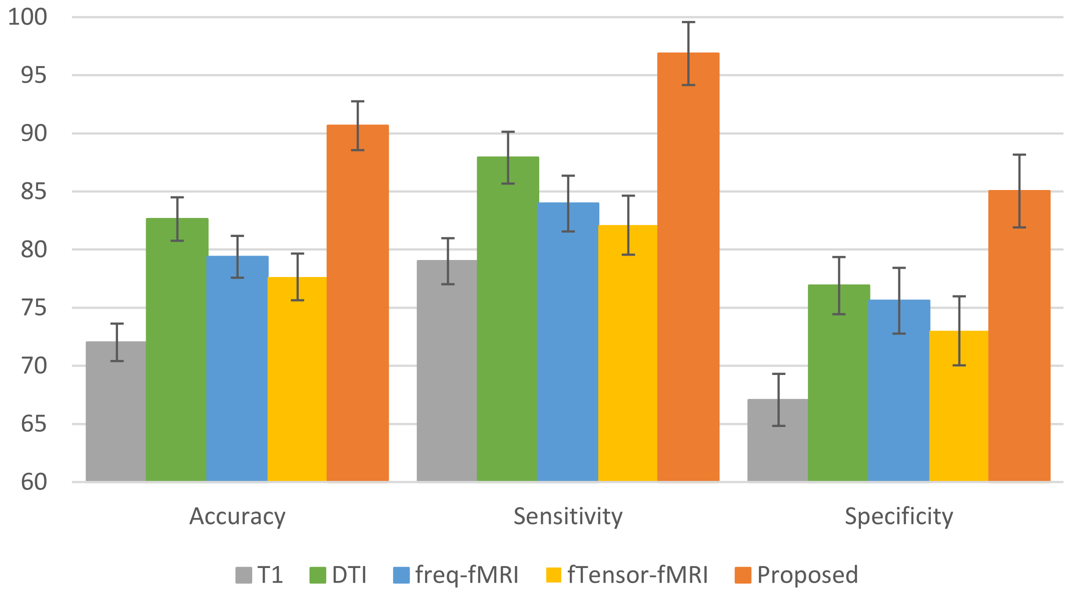

Figure 4. OS prediction results using different imaging modalities. "Proposed" denotes the prediction result by combining all modalities together.

Comparison with 2D CNN based Approaches

To illustrate the superiority of using the proposed 3D CNN architectures, we also compare our proposed method with the CNNs that use the 2D filters. Specifically, we adopted a 2D version of the CNN architecture shown in page 13, in which the inputs are 2D patches along the axial plane, and the feature maps are all reduced to 2D. Moreover, we employ the same strategy shown in page 14 to train multi-channel deep networks, with 2D features. We use slices from the tumor region ($64 \times 64 \times 64$) as input for the 2D CNNs (Note, we can also use 32x32 as the patch size to train a 2D CNN model; however, such a small patch size could not catch much information and it resulted in unfavorable results). As the dataset is small ($64 \times 68 = 4352$), we perform several fixed rotations of [90,180,270] degrees for each extracted 2D patch to augment the dataset ($4352 \times 4 = 17408$).

The experimental results are presented in Fig. 5. The 2D CNN-based approach presents a decent performance such as about 81% of accuracy, 89% of sensitivity, and 74% of specificity. In contrast, the proposed 3D CNN can advance the performance by approximately 10%. These results illustrate that the proposed 3D CNN features are more effective and powerful than the 2D-CNN based features.

Comparison with Unsupervised Feature Extraction Approaches

To show the advantage of our 3D-CNN-based supervised feature learning, we also perform comparisons with several unsupervised feature extraction techniques, which are popularly used in both computer vision and medical imaging fields.

Specifically, we adopt scale-invariant transform (SIFT)[49], a commonly used unsupervised image descriptor in image reconstruction, alignment and recognition tasks, as a comparison feature extraction approach. As our medical image is stored in 3D format, we employ a spatial-temporal descriptor based on 3D gradients[50] to extract the features from the tumor regions. We then cluster the vector-represented patches to "codewords" using k-means, which produces visual vocabularies for the features. Each patch in an image is represented by a certain visual vocabulary, and finally the image can be represented by a histogram of the visual vocabularies. This is the same procedure used in many recognition methods in computer vision, denoted as "bag of words" method[51].

We also extract the Haar-like features from tumor patches, which are originally proposed by the paper[52] for object detection and have been applied to many applications due to its efficiency. Note that we use a variant of the Haar-like features[53] calculated based on the difference between the mean values of two cubic-regions randomly located within an image patch. The size of each cubic-region is randomly chosen from an arbitrary range, i.e., 1, 3, 5 in voxels[54].

Since we have multiple modalities of data to extract the features, we first extract features from each modality separately and then use PCA to reduce their dimensionality. Next, we concatenate the features from different modalities and the handcrafted features, and finally train an SVM model. The experimental results are shown in Fig. 6. The Haar-like features present the worst performance, and the proposed deep-learning-based features result in the best performance. Specifically, our supervised feature extraction framework can improve the performance by approximately 12%.

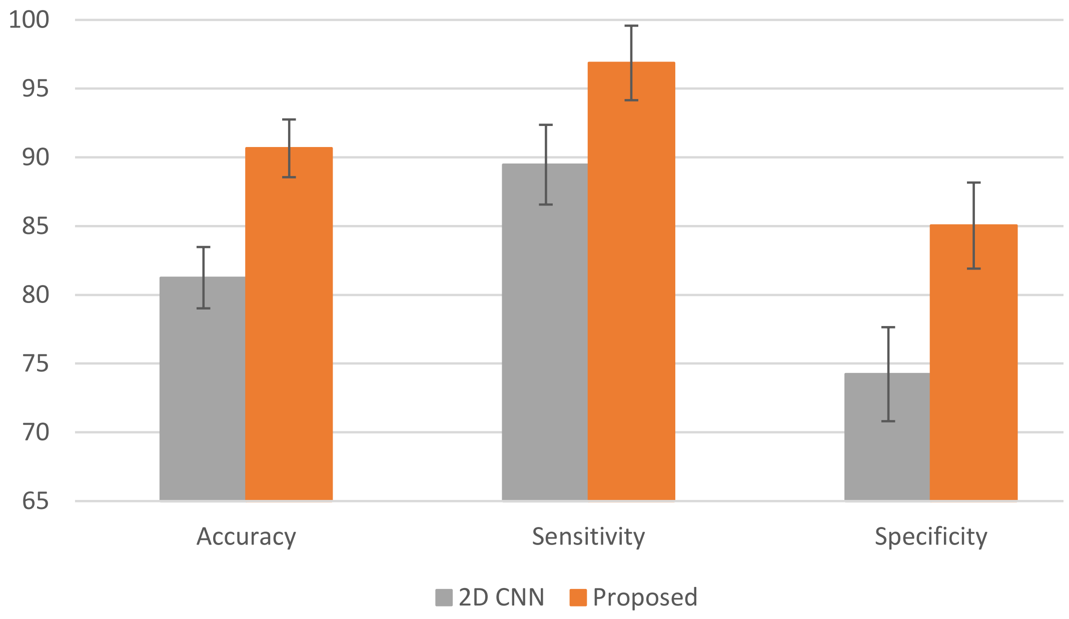

Figure 5. The experimental results using different feature extraction methods (such as 2D CNN and the proposed 3D CNN).

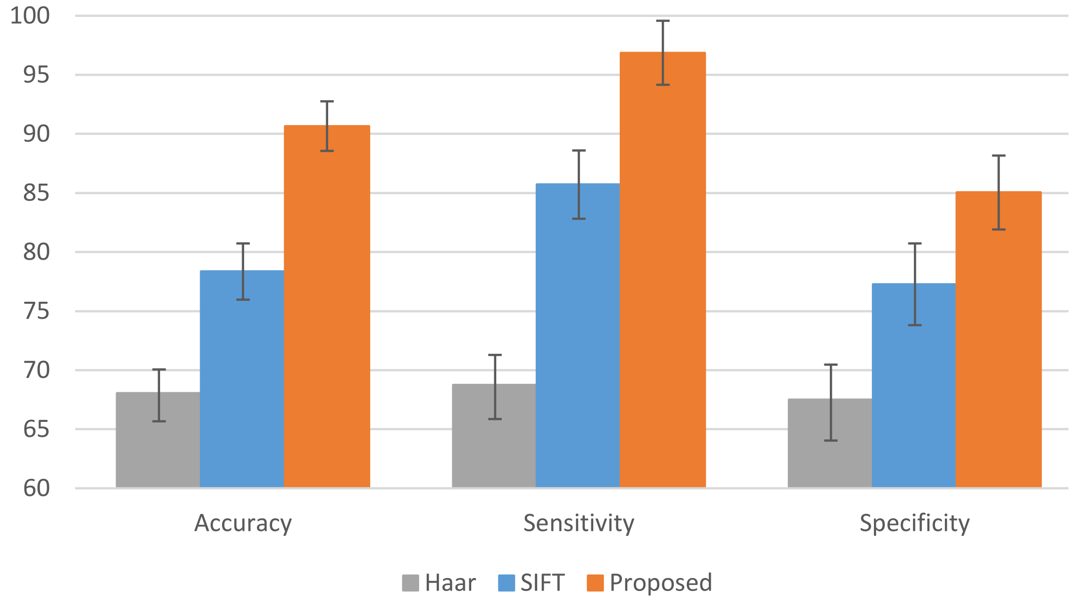

Figure 6. The experimental results using different kinds of (unsupervised vs. supervised) features.

Impact of Combining CNN and SVM

As reported in the Method Section, our proposed method combines CNN and SVM together, since we assume that CNN can well learned semantic features in a patch-level, and SVM can well handle a small samples-size classification problem in subject-level. To investigate the impact of combining these two models, we design comparison experiments with SVM alone method and CNN alone method.

With SVM alone, we used manual designed features, i.e., Haar and SIFT features, as reported in the Section "Comparison with Unsupervised Feature Extraction Approaches". As for CNN-based classification, as CNN has an ability to combine feature learning and classification together, which directly generates the soft label as the final output from the neuronal network. Therefore, one can directly use CNN's output (by treating the largest soft label result as the final classification result) as the OS prediction result. The rationale that we design our model (CNN-based feature extraction plus SVM-based classification) is that

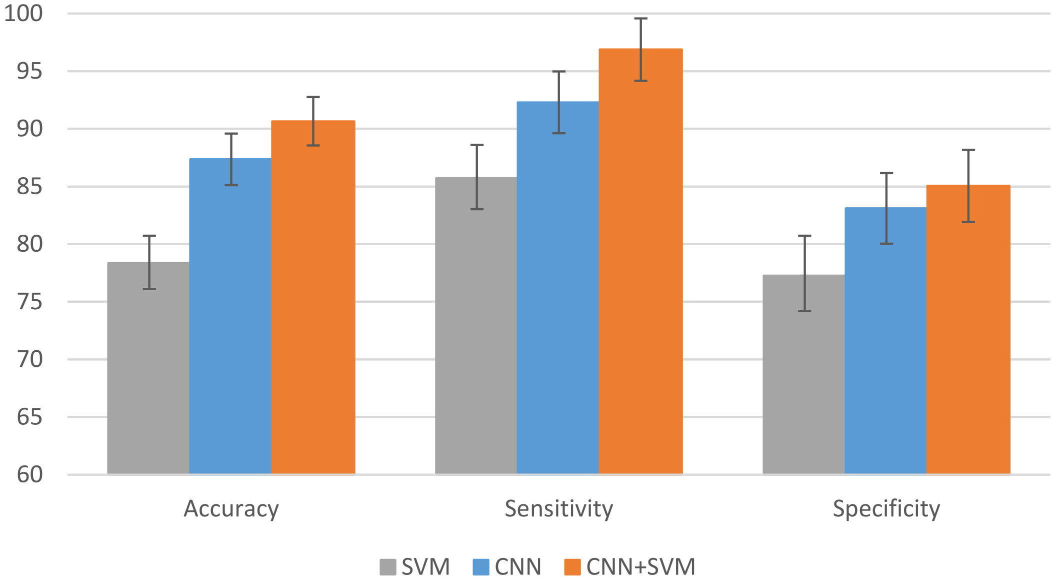

Figure 7. Comparison of experimental results with SVM, CNN and CNN+SVM.

SVM generally performs better and more robustly in a study with limited sample size. Therefore, we designed CNN+SVM by extracting features from the fully connected layers as the inputs of SVM for classification.

To make the comparison results easier to understand, we concluded our results from using CNN alone, using SVM alone (with SIFT features) and using CNN+SVM in Fig. 7. CNN alone performs better than SVM alone, as CNN can learn better feature representations. Since the CNN+SVM conduct the classification on the subject-level feature representations which can well aggregate the patch-level features, it can improve the performance about 2.5% compared to CNN alone.

Model Transparency and Robustness

Deep learning based models hierarchically process the input data (imaging data in our case), and output the highly semantic features towards the target (i.e., tumor OS prediction in our case). As in our study, we use the last two layers (i.e., fc6 and fc7) as the extracted features. These features will be highly semantic and quite effective for tumor OS classification as they are learned under supervision. However, it is currently difficult to investigate which imaging features really help improve the accuracy and what they really represent. It is also difficult for our study, although we have tried to investigate which region of the imaging data contributes most to the useful features.

However, the lack of transparency doesn't affect the robustness of our model. As reported in Section **Experiments and Results**, we first validate our proposed method on the dataset with 68 patients in a 3-fold cross-validation fashion. Then we further validate it by introducing extra testing on a new dataset with 25 patients. The experimental results on these two datasets indicate that our proposed method is robust. Furthermore, a lot of similar researches based on deep learning models are recently proposed and achieve great success. For example, Setio et al. proposed a multi-stream CNN to categorize the points of interest in chest CT as a nodule or non-nodule[55]. Esteva et al. proposed a deep neural network to implement dermatologist-level classification of skin cancer[56]. And part of these studies has even been applied to clinical trials. Thus, we believe our proposed method is useful in developing a new tumor OS prediction model.

Limitation and Future Works

It is worth indicating the limitations of our work. For example, we only use limited clinical information in our study and thus obtain a weak clinical model. We have also thought of using other features such as genetic indicators for OS prediction[8], including IDH1, MGMT, EGFR, 1p19q. The newly revised WHO grading system even suggests using some of the genetic features to grade the gliomas. Unfortunately, we did not have such information for all the subjects because our data were collected several years ago (at that time, collecting genetic information has not become the clinical routine yet). On the other hand, in our recent paper[57], we have used our newly-enrolled subjects' neuroimaging data to predict their genotype information (IDH1 and MGMT), indicating the existence of relationship between imaging phenotypes and genotypes. But, for these newly-enrolled subjects, since they were newly admitted to the hospital and have been only followed up for a short time, we have not had their OS information yet. In the future follow-up study, as more subjects with both genetic information and OS data, we will include genetic information for OS prediction.

As for other important predictive features related to treatment, such as the extent of resection and the type/dose/duration of the adjuvant therapy, we did check this information before carrying on this study. However, as mentioned in the Introduction section, the motivation of this study is to answer a question: “Can we predict the patients’ OS based on their presurgical neuroimages, given similar treatments”. Therefore, in the experimental design, we have deliberately enrolled subjects with total (or gross total) resection; for most of them, they have been conducted with postsurgical adjuvant radiotherapy and chemotherapy with the same protocol suggested by the guideline. With these specifically selected subjects, we can then reduce the confounding effect of treatment and focus more on the prognostic value of neuroimaging. In post hoc analysis, we found that there is no group difference in extension of resection ($p = 0.22$) and postoperative treatments ($p = 0.82$) between short and long OS groups. Of note, we acknowledge that treatment is very important to OS, and we are carrying on an ongoing study to predict OS based on both presurgical and treatment features, as well as genetic features, so that future treatment can be better tailored for each individual.

As discussed earlier, our experiments are conducted on a dataset with 68 subjects and a new dataset with 25 patients. The number of subjects is relatively small. Therefore, to obtain better generalizability of the proposed method, we need to increase the participating subjects in the future. Also, we simply concatenate features (fc6 or fc7) extracted from different modalities together and utilize them for subsequent OS prediction without considering the relationship between different modalities. We should better take it into consideration in the future work. Moreover, we have resized the tumor cuboids to make them consistent in size; however, this operation obviously affects parts of the geometric properties of the tumor. This issue can be possibly resolved by applying a multi-instance learning framework. Furthermore, our current model considers tumor patients of WHO III and IV together, and we can potentially build separate models for WHO III and WHO IV patients to make more detailed predictions. Lastly, we choose a hard threshold to classify the patients into two categories (long or short OS), which decreases the precision of our predictive results. Besides, we can further categorize the patients into more (e.g., 4 or 5) subgroups for making more precise predictions.

In our future work, we will use all currently available features, including features from presurgical imaging, treatment ways, patient statuses before and after surgery, genetic information and molecular indicators (including IDH1, MGMT, 1p19q, TERT, and ATRX), to perform OS prediction. Since these features are from different domains, more advanced feature learning and integration methods need to be developed. Moreover, since our ultimate goal is to predict the overall survival which can be better used in clinical practice, we will treat it as continuous variable with a rigorous machine-learning or deep-learning-based regression model in our future work.

Generally, in this study, we have proposed a 3D deep learning model to predict the (long or short) OS time for the patients with brain glioma. We trained 3D CNN and mCNN models for learning features from single-channel (T1 MRI) and multi-channel (DTI, freq-fMRI and fTensor-fMRI) data in a supervised manner, respectively. The extracted features were then fed into a binary SVM classifier. The performance of our supervised CNN-based learned features was compared with the performances of several other state-of-the-art methods, including those using the traditional handcrafted features. Experimental results showed that our supervised-learned features significantly improved the predictive accuracy of OS time for the glioma patients. This also indicates that our proposed 3D deep learning frameworks can provoke computational models to extract useful features for such neuro-oncological applications. Besides, the analysis on the selected features further shows that DTI data can contribute slightly more than fMRI, but both fMRI and DTI play more significant role, compared to the T1 MRI, in building successful prediction models. Overall, our proposed method shows its great promise in multi-modal MRI-based diagnosis or prognosis for a wider spectrum of neurological and psychiatric diseases.

Method

In this paper, we first employ the CNN architecture to train survival prediction models with the patches from all metric maps of T1 MRI, DTI and rs-fMRI, respectively. With such trained deep learning models, we can extract features for individual patches of the respective channels/modalities in a supervised manner. Then, a binary classifier (i.e., SVM) is trained to fuse all the patches and their extracted high-level features for OS prediction (Fig. 1). In the following subsections, we will introduce both our CNN-based feature extraction and the SVM-based OS prediction strategies. Note that the CNN-based feature extraction for T1 MRI is slightly different from that for DTI and fMRI. That is, there is only a single inputting channel for T1 MRI, while there are multiple inputting channels for multiple metrics computed from DTI and fMRI. Thus, we will first introduce the 3D CNN architecture for single-channel T1 MRI, and then extend it for multi-channel DTI and fMRI. Different from the conventional CNN that stacks multi-channel inputs at the beginning, we perform independent convolution streams for each inputting channel in the early layers and then fuse them in deep layers for high-level feature extraction. Of note, to augment the dataset, we flip the bounding box along three directions (x, y, z) separately for all metrics. Next, we extract numerous partially-overlapping patches with the size of $32 \times 32 \times 32$ to train the CNNs.

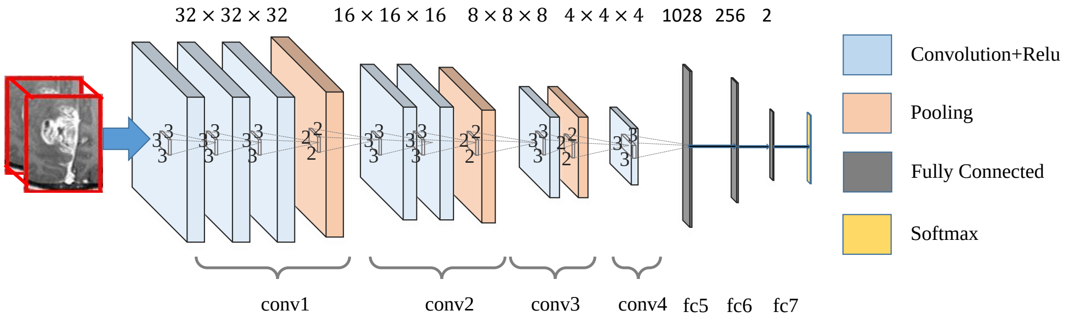

Figure 8. An illustration of the CNN architecture for the single-channel feature extraction from 3D patches. There are four convolutional layer groups and three fully-connected layers. The network's input is a $32 \times 32 \times 32$ patch from a tumor region of a patient, and the output is the decision that this patient belongs to the long or short survival group.

Single-Channel Feature Extraction

For 3D T1 MRI, we propose a 3D CNN model with a set of 3D trainable filters. CNN derives the high-level features from the low-level input, while the estimated high-level features directly contribute to the classification of the input data. The network architecture usually consists of a number of layers. As we go deeper in the network, the layer will generate higher-level features. For example, the last layer can represent more intrinsic features compared to the earlier layer(s)[58].

Inspired by the very deep convolutional networks (VGGNet)[59], we design our CNN architecture with four convolutional layer groups and three fully-connected layers. The detailed configurations of the four convolutional layer groups (conv1 to conv4) are shown in Fig. 8. The input to the CNN is a 3D patch with the size of $32 \times 32 \times 32$, which is extracted in the bounded neighborhood of the tumor. The convolutional layers compute their outputs from the input 3D patch, by applying the convolutional operations with 3D filters of the size $3 \times 3 \times 3$. The convolutional operation results in the 3D output patch of the same size as the input, followed by max-pooling to down-sample the patch. The last three layers in the CNN are fully connected (fc5 to fc7). These fully-connected layers include the neurons that are connected to all outputs of their precedent layers, as in the conventional neural networks. The last layer (fc7) has 2 neurons, whose correspond to the probabilities of classifying the patient into the long or the short OS group.

The supervision on the classification of the training data leads to a back-propagation procedure for learning the most relevant features in the CNN. Specifically, we regard the outputs from the last two layers of the CNN (fc6 and fc7) as the learned high-level appearance features of individual input patch. The efficiency and effectiveness of the extracted features will be verified in the experiments.

There are four convolutional layer groups and three fully-connected layers. The network's input is a $32 \times 32 \times 32$ patch from a tumor region of a patient, and the output is the decision that this patient belongs to the long or short survival group.

Multi-Channel Feature Extraction

We compute multiple metric maps for DTI and rs-fMRI. Each metric map corresponds to an input channel when learning the high-level appearance features. To effectively employ all multi-channel data for providing complementary information for the brain tumor, we propose a new multi-channel-CNN (mCNN) architecture to train one mCNN for each modality. Inspired by the multi-modal deep Boltzmann machine[60], we extend our single-channel 3D CNN architecture to deal with multi-channel data. Specifically, in the proposed mCNN, the same convolutional layer groups are applied to each channel separately. Then, a fusion layer is added to integrate the outputs of the last convolutional layer group (conv4) from all channels by concatenating them. Then, three fully-connected layers are further incorporated to finally extract the features. The mCNN architecture is illustrated in Fig. 9. Note that the major difference between mCNN and single-channel CNN is the fusion layer. Other layers, including the convolutional layers and the fully-connected layers, follow the same configuration. It is important to note that the statistical properties of different channels of the input data can vary largely, which makes it difficult for a single-channel model to directly encode multi-channel data (i.e., by simply concatenating multi-channel data and then applying the single-channel model). In contrast, our proposed mCNN model sustains much better capability of modeling multi-channel input data and fusing them together to generate high-level features.

SVM-Based Survival Prediction

Once we complete training a CNN (Fig. 8) for T1 MRI and two mCNN (Fig. 9 for DTI and fMRI, respectively, we can predict the short/long survival given 3D patches extracted in each of the four "modalities" (note that we derive two sub-modalities from rs-fMRI, i.e., freq-fMRI and fTensor-fMRI). That is, the patch(es), from single or multiple channels of the metrics, will

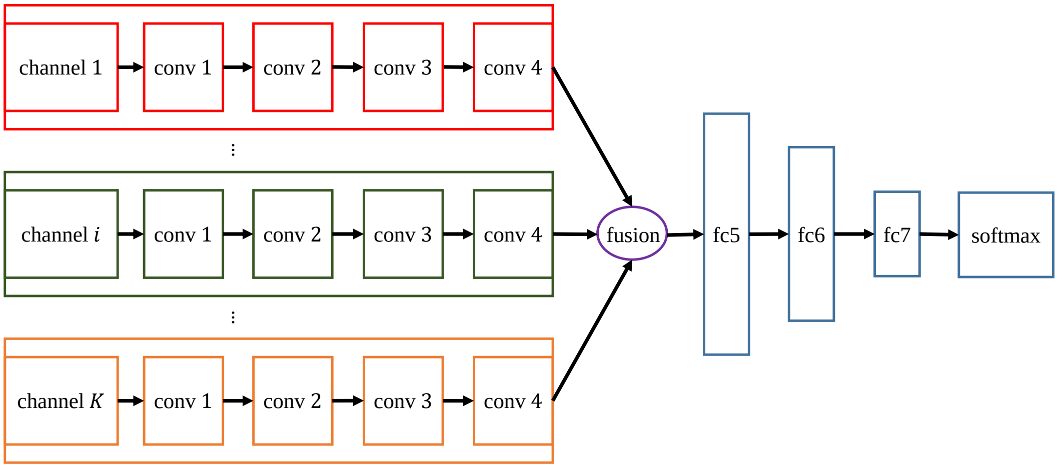

Figure 9. Architecture of mCNN for feature extraction from multi-channel data.

go through the convolutional networks. Then the CNNs convert the input patch(es) to the high-level features and obtain the survival estimation in the final layer. In particular, the high-level features extracted at the last two layers (fc6 and fc7) of our CNN architectures are perceived to be suitable image-level descriptors[61]. In this way, each patch can associate its high-level features with the survival time of the patient under consideration. Note that the fc6 layer has 256 neurons, while the last (fc7) layer comprises of two neurons.

In addition to these high-level appearance features, the limited demographic and tumor-related features (dtf) are also included in our experiments. These limited demographic and tumor-related features consist of generic brain tumor features, including gender, age at diagnosis, tumor location, size of tumor, and the WHO grade. Tumor location is defined by two metrics, i.e., 1) major location (such as the brain lobe where the tumor is mainly located), and 2) tumor distribution (i.e., a three-grade scale, ranging from 1 to 3, denoting the number of different brain lobes (such as 5 different lobes used in this study, including occipital, temporal, parietal, frontal and insula lobe) with tumor). For example, the tumor distribution of 1 denotes that the tumor appears only in one brain lobe. The assessment was conducted by three authors (HZ, JW and JL) with consensus. The size of the tumor was calculated based on T1 contrast-enhanced MRI by manually delineating the volume with abnormal intensity (i.e., the volume of a tumor as shown in the presurgical imaging). This was conducted by one neurosurgeon with 8-year experience (JL) to ensure the consistent tumor delineation criteria. There is no significant difference in the tumor volume ($p = 0.55$) between the two OS groups. Patient performance status was evaluated by using Karnofsky Performance Status (KPS) scores; however, the two OS groups have no group difference in KPS score ($p > 0.99$). For the treatment variables, between the two OS groups, there is no significant difference in resection extension ($p = 0.22$), and the ways of post-surgical treatments received ($p = 0.82$). Although not included as the limited demographic and tumor-related features, we think that these factors will not likely to make significant contribution to the individualized OS prediction.

Since the numbers of the features in the fc6 and fc7 layers are huge, we further conduct feature reduction or selection for the features from each modality separately. Specifically, we use Principal Component Analysis (PCA) and Sparse Representation (SR)[47] (see **Experimental Settings**). Finally, we adopt SVM[35] to predict the patient's survival time at an individual level based on the selected features.

References

1. Curran Jr, W. J. *et al.* Recursive partitioning analysis of prognostic factors in three radiation therapy oncology group malignant glioma trials. *JNCI: J. Natl. Cancer Inst.* **85**, 704–710 (1993).

2. Gittleman, H. *et al.* An independently validated nomogram for individualized estimation of survival among patients with newly diagnosed glioblastoma: Nrg oncology rtog 0525 and 0825. *Neuro-oncology* **19**, 669–677 (2017).

3. Lacroix, M. *et al.* A multivariate analysis of 416 patients with glioblastoma multiforme: prognosis, extent of resection, and survival. *J. neurosurgery* **95**, 190–198 (2001).

4. DeAngelis, L. M. Brain tumors. *New Engl. J. Medicine* **344**, 114–123 (2001).

5. Guillamo, J.-S. *et al.* Brainstem gliomas in adults: prognostic factors and classification. *Brain* **124**, 2528–2539 (2001).

6. Coons, S. W., Johnson, P. C., Scheithauer, B. W., Yates, A. J. & Pearl, D. K. Improving diagnostic accuracy and interobserver concordance in the classification and grading of primary gliomas. *Cancer* **79**, 1381–1393 (1997).

7. Eckel-Passow, J. E. *et al.* Glioma groups based on 1p/19q, idh, and tert promoter mutations in tumors. *New Engl. J. Medicine* **372**, 2499–2508 (2015).

8. Network, C. G. A. R. Comprehensive, integrative genomic analysis of diffuse lower-grade gliomas. *New Engl. J. Medicine* **372**, 2481–2498 (2015).

9. van den Bent, M. J. Interobserver variation of the histopathological diagnosis in clinical trials on glioma: a clinician's perspective. *Acta neuropathologica* **120**, 297–304 (2010).

10. Itakura, H. *et al.* Magnetic resonance image features identify glioblastoma phenotypic subtypes with distinct molecular pathway activities. *Sci. translational medicine* **7**, 303ra138–303ra138 (2015).

11. Zhang, L. *et al.* Learning-based structurally-guided construction of resting-state functional correlation tensors. *Magn. resonance imaging* **43**, 110–121 (2017).

12. Gutman, D. A. *et al.* Mr imaging predictors of molecular profile and survival: multi-institutional study of the tcga glioblastoma data set. *Radiol.* **267**, 560–569 (2013).

13. Kickingereder, P. *et al.* Radiomic profiling of glioblastoma: identifying an imaging predictor of patient survival with improved performance over established clinical and radiologic risk models. *Radiol.* **280**, 880–889 (2016).

14. Wang, Y. *et al.* Patterns of tumor contrast enhancement predict the prognosis of anaplastic gliomas with idh1 mutation. *Am. J. Neuroradiol.* **36**, 2023–2029 (2015).

15. Pope, W. B. *et al.* Mr imaging correlates of survival in patients with high-grade gliomas. *Am. J. Neuroradiol.* **26**, 2466–2474 (2005).

16. Jain, R. *et al.* Genomic mapping and survival prediction in glioblastoma: molecular subclassification strengthened by hemodynamic imaging biomarkers. *Radiol.* **267**, 212–220 (2013).

17. Jain, R. *et al.* Outcome prediction in patients with glioblastoma by using imaging, clinical, and genomic biomarkers: focus on the nonenhancing component of the tumor. *Radiol.* **272**, 484–493 (2014).

18. Reyes-Botero, G. *et al.* Contrast enhancement in 1p/19q-codeleted anaplastic oligodendrogliomas is associated with 9p loss, genomic instability, and angiogenic gene expression. *Neuro-oncology* **16**, 662–670 (2013).

19. Saksena, S. *et al.* Predicting survival in glioblastomas using diffusion tensor imaging metrics. *J. Magn. Reson. Imaging* **32**, 788–795 (2010).

20. Oh, J. *et al.* Survival analysis in patients with glioblastoma multiforme: Predictive value of choline-to-n-acetylaspartate index, apparent diffusion coefficient, and relative cerebral blood volume. *J. Magn. Reson. Imaging* **19**, 546–554 (2004).

21. Zacharaki, E. I. *et al.* Survival analysis of patients with high-grade gliomas based on data mining of imaging variables. *Am. J. Neuroradiol.* **33**, 1065–1071 (2012).

22. Zhang, D. *et al.* Preoperative sensorimotor mapping in brain tumor patients using spontaneous fluctuations in neuronal activity imaged with functional magnetic resonance imaging: initial experience. *Oper. Neurosurg.* **65**, ons226–ons236 (2009).

23. Lee, S.-P., Duong, T. Q., Yang, G., Iadecola, C. & Kim, S.-G. Relative changes of cerebral arterial and venous blood volumes during increased cerebral blood flow: implications for bold fmri. *Magn. resonance medicine* **45**, 791–800 (2001).

24. Pillai, J. J. & Mikulis, D. Cerebrovascular reactivity mapping: an evolving standard for clinical functional imaging. *Am. J. Neuroradiol.* **36**, 7–13 (2015).

25. Hardee, M. E. & Zagzag, D. Mechanisms of glioma-associated neovascularization. *The Am. journal pathology* **181**, 1126–1141 (2012).

26. Agnihotri, S. & Zadeh, G. Metabolic reprogramming in glioblastoma: the influence of cancer metabolism on epigenetics and unanswered questions. *Neuro-oncology* **18**, 160–172 (2015).

27. Mineo, J.-F. *et al.* Prognosis factors of survival time in patients with glioblastoma multiforme: a multivariate analysis of 340 patients. *Acta neurochirurgica* **149**, 245–253 (2007).

28. Cui, Y. *et al.* Prognostic imaging biomarkers in glioblastoma: development and independent validation on the basis of multiregion and quantitative analysis of mr images. *Radiol.* **278**, 546–553 (2015).

29. Glorot, X. & Bengio, Y. Understanding the difficulty of training deep feedforward neural networks. In *Proceedings of the Thirteenth International Conference on Artificial Intelligence and Statistics*, 249–256 (2010).

30. LeCun, Y., Bengio, Y. & Hinton, G. Deep learning. *nature* **521**, 436 (2015).

31. LeCun, Y., Bottou, L., Bengio, Y. & Haffner, P. Gradient-based learning applied to document recognition. *Proc. IEEE* **86**, 2278–2324 (1998).

32. Shen, D., Wu, G. & Suk, H.-I. Deep learning in medical image analysis. *Annu. review biomedical engineering* **19**, 221–248 (2017).

33. Spanhol, F. A., Oliveira, L. S., Petitjean, C. & Heutte, L. Breast cancer histopathological image classification using convolutional neural networks. In *Neural Networks (IJCNN), 2016 International Joint Conference on*, 2560–2567 (IEEE, 2016).

34. Chang, C.-C. & Lin, C.-J. Libsvm: a library for support vector machines. *ACM transactions on intelligent systems technology (TIST)* **2**, 27 (2011).

35. Fan, R.-E., Chang, K.-W., Hsieh, C.-J., Wang, X.-R. & Lin, C.-J. Liblinear: A library for large linear classification. *J. machine learning research* **9**, 1871–1874 (2008).

36. Nie, D., Zhang, H., Adeli, E., Liu, L. & Shen, D. 3d deep learning for multi-modal imaging-guided survival time prediction of brain tumor patients. In *International Conference on Medical Image Computing and Computer-Assisted Intervention*, 212–220 (Springer, 2016).

37. Wu, J.-S. *et al.* Clinical evaluation and follow-up outcome of diffusion tensor imaging-based functional neuronavigation: a prospective, controlled study in patients with gliomas involving pyramidal tracts. *Neurosurg.* **61**, 935–949 (2007).

38. Stupp, R. *et al.* Promising survival for patients with newly diagnosed glioblastoma multiforme treated with concomitant radiation plus temozolomide followed by adjuvant temozolomide. *J. Clin. Oncol.* **20**, 1375–1382 (2002).

39. Cui, Z., Zhong, S., Xu, P., Gong, G. & He, Y. Panda: a pipeline toolbox for analyzing brain diffusion images. *Front. human neuroscience* **7**, 42 (2013).

40. Alexander, A. L. *et al.* Characterization of cerebral white matter properties using quantitative magnetic resonance imaging stains. *Brain connectivity* **1**, 423–446 (2011).

41. Yan, C. & Zang, Y. Dparsf: a matlab toolbox for" pipeline" data analysis of resting-state fmri. *Front. systems neuroscience* **4**, 13 (2010).

42. Buzsáki, G. & Draguhn, A. Neuronal oscillations in cortical networks. *science* **304**, 1926–1929 (2004).

43. Penttonen, M. & Buzsáki, G. Natural logarithmic relationship between brain oscillators. *Thalamus & Relat. Syst.* **2**, 145–152 (2003).

44. Ding, Z. *et al.* Visualizing functional pathways in the human brain using correlation tensors and magnetic resonance imaging. *Magn. resonance imaging* **34**, 8–17 (2016).

45. Wu, T.-L. *et al.* Effects of anesthesia on resting state bold signals in white matter of non-human primates. *Magn. resonance imaging* **34**, 1235–1241 (2016).

46. Jia, Y. *et al.* Caffe: Convolutional architecture for fast feature embedding. In *Proceedings of the 22nd ACM international conference on Multimedia*, 675–678 (ACM, 2014).

47. Guyon, I. & Elisseeff, A. An introduction to variable and feature selection. *J. machine learning research* **3**, 1157–1182 (2003).

48. Liu, J., Ji, S., Ye, J. *et al.* Slep: Sparse learning with efficient projections. *Ariz. State Univ.* **6**, 7 (2009).

49. Lowe, D. G. Distinctive image features from scale-invariant keypoints. *Int. journal computer vision* **60**, 91–110 (2004).

50. Scovanner, P., Ali, S. & Shah, M. A 3-dimensional sift descriptor and its application to action recognition. In *Proceedings of the 15th ACM international conference on Multimedia*, 357–360 (ACM, 2007).

51. Yang, J., Jiang, Y.-G., Hauptmann, A. G. & Ngo, C.-W. Evaluating bag-of-visual-words representations in scene classification. In *Proceedings of the international workshop on Workshop on multimedia information retrieval*, 197–206 (ACM, 2007).

52. Viola, P. & Jones, M. J. Robust real-time face detection. *Int. journal computer vision* **57**, 137–154 (2004).

53. Zhang, L., Wang, Q., Gao, Y., Wu, G. & Shen, D. Automatic labeling of mr brain images by hierarchical learning of atlas forests. *Med. physics* **43**, 1175–1186 (2016).

54. Zhang, L. *et al.* Concatenated spatially-localized random forests for hippocampus labeling in adult and infant mr brain images. *Neurocomputing* **229**, 3–12 (2017).

55. Setio, A. A. A. *et al.* Pulmonary nodule detection in ct images: false positive reduction using multi-view convolutional networks. *IEEE transactions on medical imaging* **35**, 1160–1169 (2016).

56. Esteva, A. *et al.* Dermatologist-level classification of skin cancer with deep neural networks. *Nat.* **542**, 115 (2017).

57. Chen, L. *et al.* Multi-label inductive matrix completion for joint mgmt and idh1 status prediction for glioma patients. In *International Conference on Medical Image Computing and Computer-Assisted Intervention*, 450–458 (Springer, 2017).

58. Xu, Y. *et al.* Deep learning of feature representation with multiple instance learning for medical image analysis. In *Acoustics, Speech and Signal Processing (ICASSP), 2014 IEEE International Conference on*, 1626–1630 (IEEE, 2014).

59. Simonyan, K. & Zisserman, A. Very deep convolutional networks for large-scale image recognition. *arXiv preprint arXiv:1409.1556* (2014).

60. Srivastava, N. & Salakhutdinov, R. R. Multimodal learning with deep boltzmann machines. In *Advances in neural information processing systems*, 2222–2230 (2012).

61. Long, J. L., Zhang, N. & Darrell, T. Do convnets learn correspondence? In *Advances in Neural Information Processing Systems*, 1601–1609 (2014).

Acknowledgements (not compulsory)

This work was supported by National Institutes of Health (NIH) grants (EB006733, EB008374, MH100217, MH108914, AG041721, AG049371, AG042599, AG053867, EB022880).

Author contributions statement

D.N. and H.Z. conducted the experiments. J.L., Z.Y., L.L. and J.W. processed the data. D.N., H.Z., J.W. and D.S. wrote and revised the main manuscript text. All authors reviewed the manuscript.

Competing interests

The authors declare no competing interests.
