# Supplementary figures and images for: Multi-Channel 3D Deep Feature Learning for Survival Time Prediction of Brain Tumor Patients Using Multi-Modal Neuroimages
Source: Sci Rep. 2019 Jan 31;9:1103. doi: 10.1038/s41598-018-37387-9 (PMC6355868; doi:10.1038/s41598-018-37387-9)

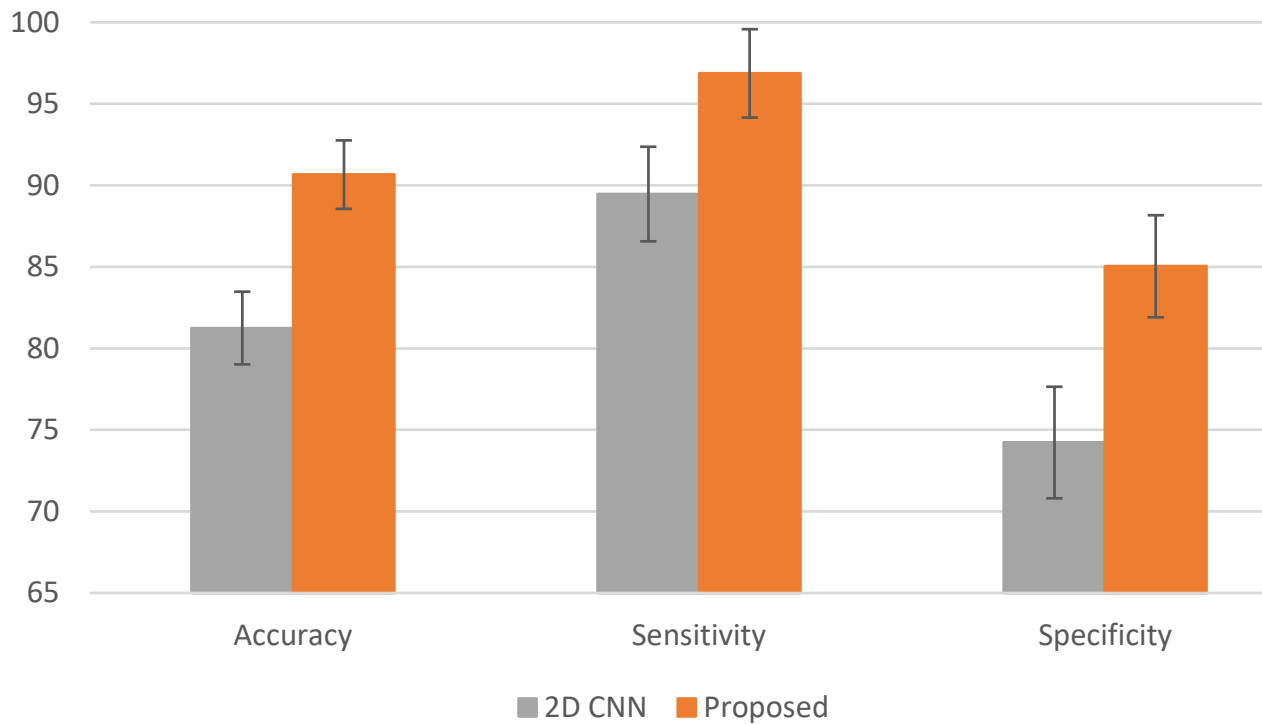

Supplement: Supplementary file 1 — LaTeX Supplementary File [file 41598_2018_37387_MOESM1_ESM.zip › Fig/2DComparison.pdf]

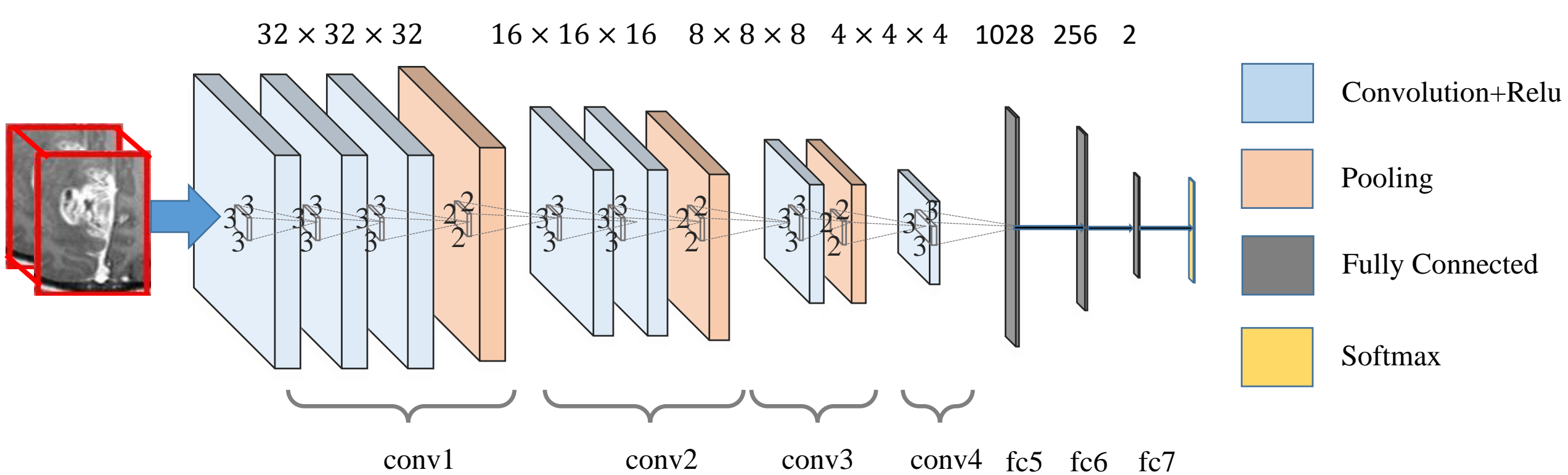

Supplement: Supplementary file 1 — LaTeX Supplementary File [file 41598_2018_37387_MOESM1_ESM.zip › Fig/cnnArchs_vivid.pdf]

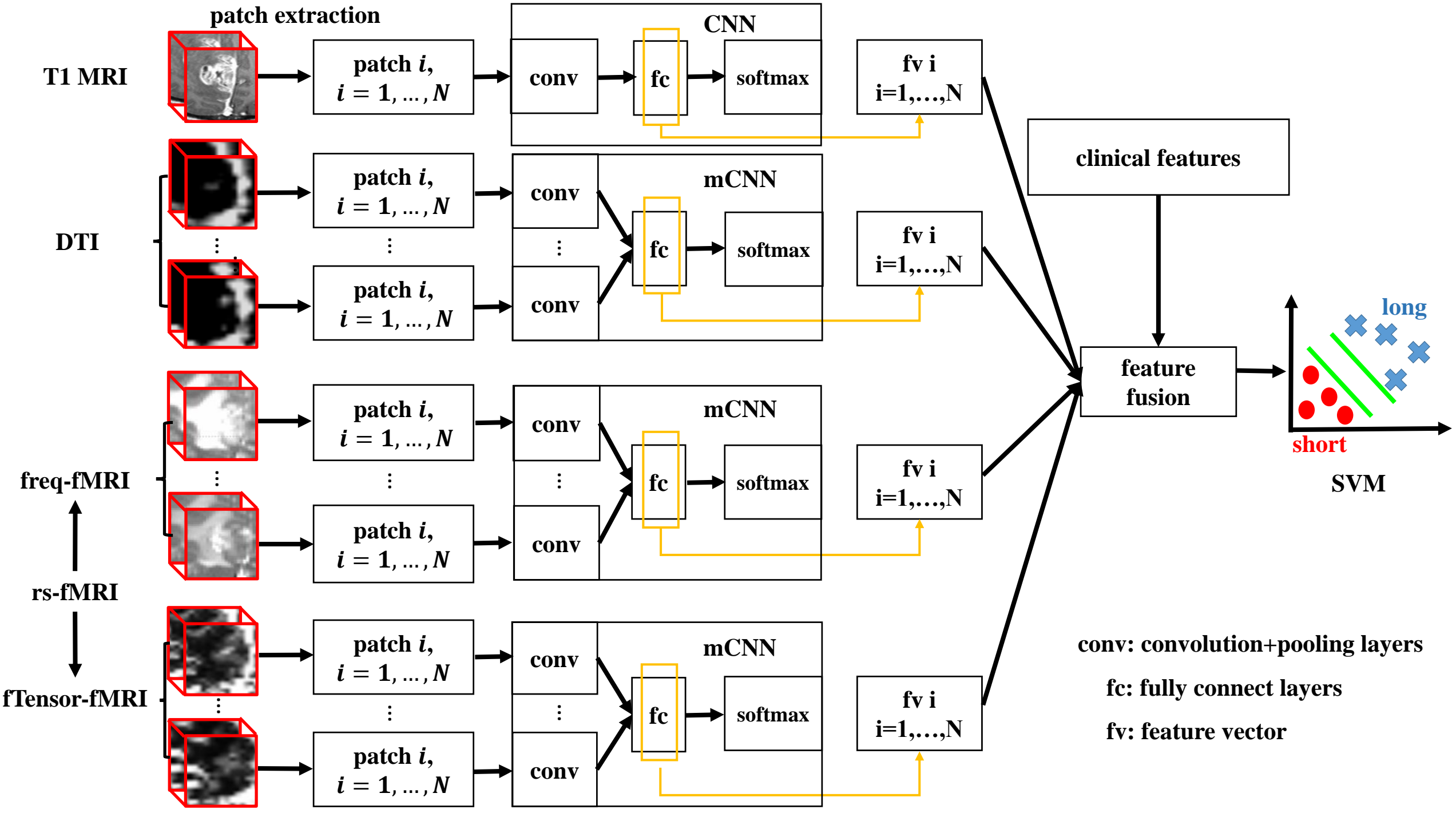

Supplement: Supplementary file 1 — LaTeX Supplementary File [file 41598_2018_37387_MOESM1_ESM.zip › Fig/flowchart_new2.pdf]

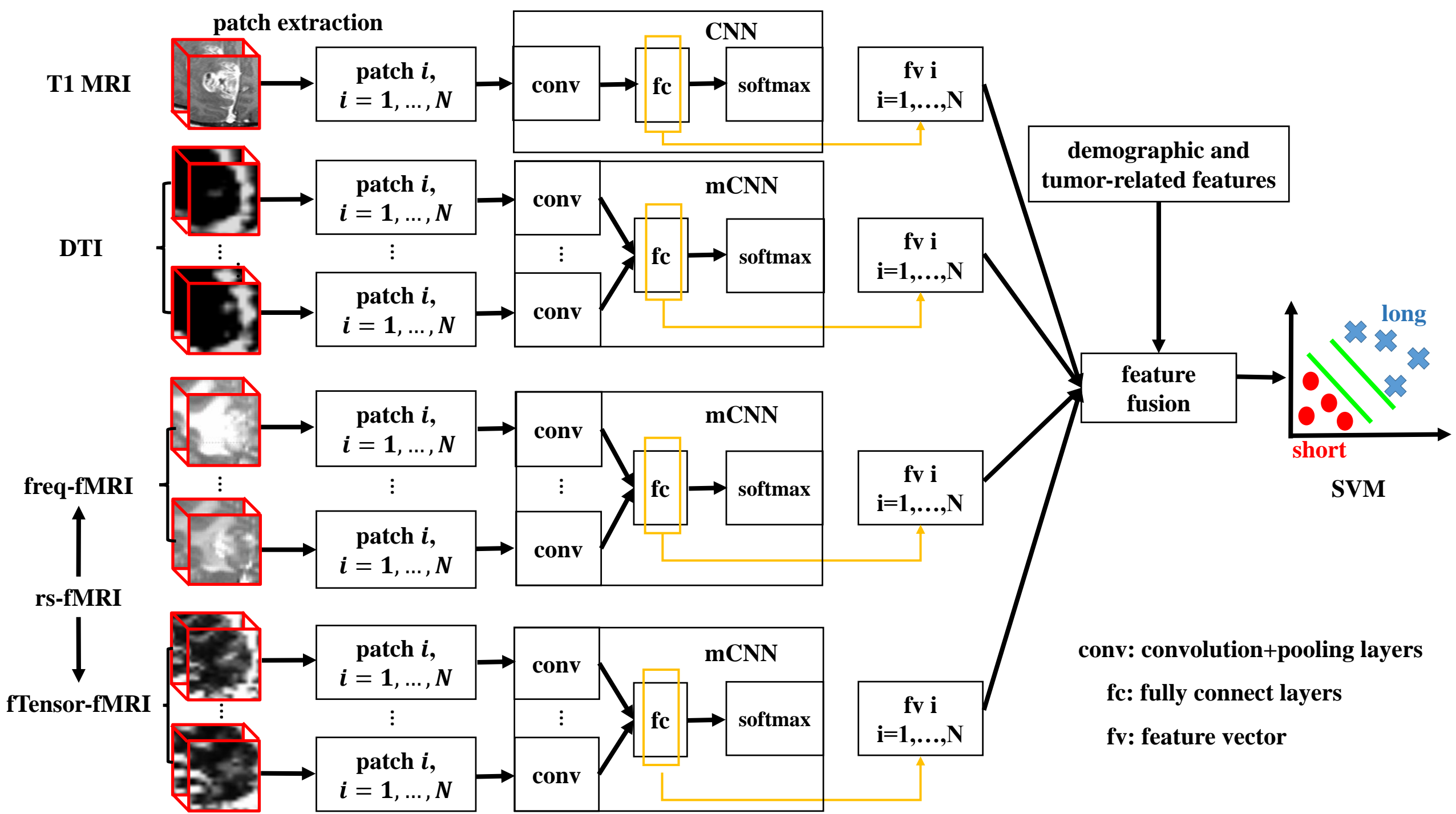

Supplement: Supplementary file 1 — LaTeX Supplementary File [file 41598_2018_37387_MOESM1_ESM.zip › Fig/flowchart_vivid_0804.pdf]

# Survival Curve based on Our Prediction Model

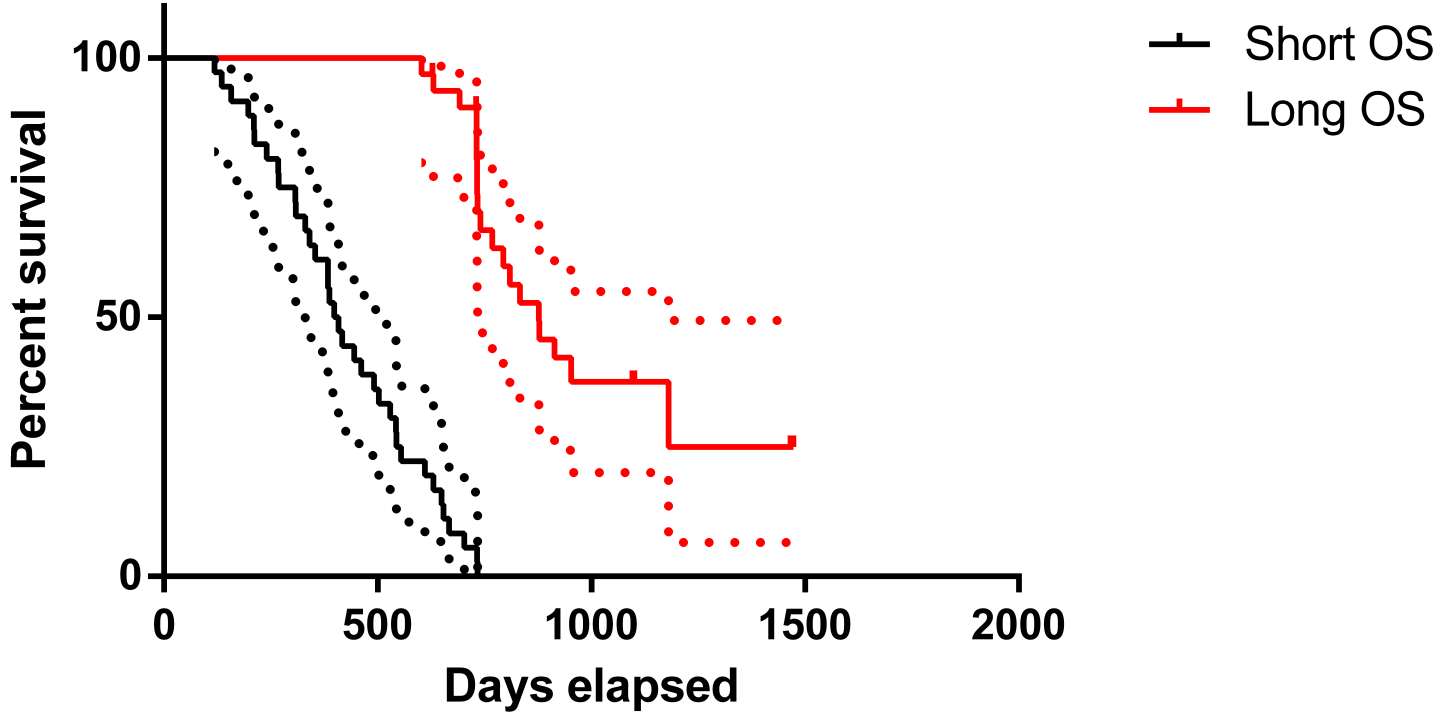

Supplement: Supplementary file 1 — LaTeX Supplementary File [file 41598_2018_37387_MOESM1_ESM.zip › Fig/km_plot.pdf]

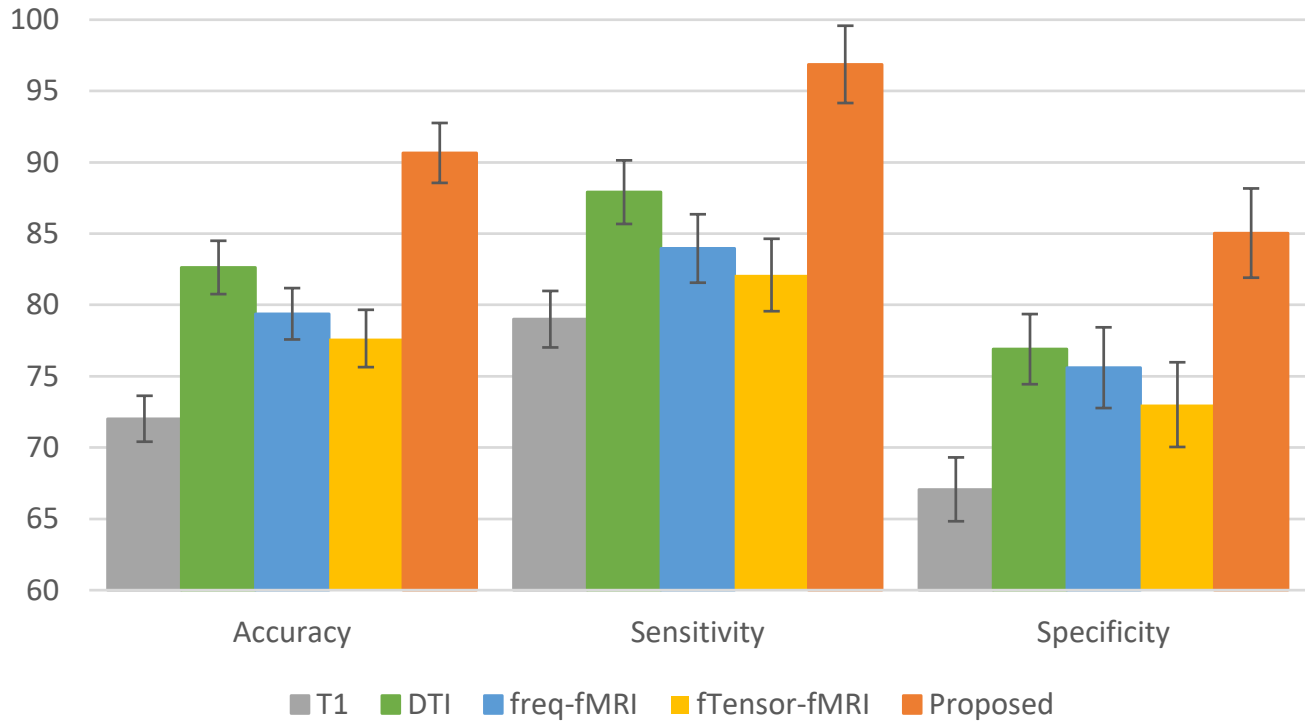

Supplement: Supplementary file 1 — LaTeX Supplementary File [file 41598_2018_37387_MOESM1_ESM.zip › Fig/modalityComparison.pdf]

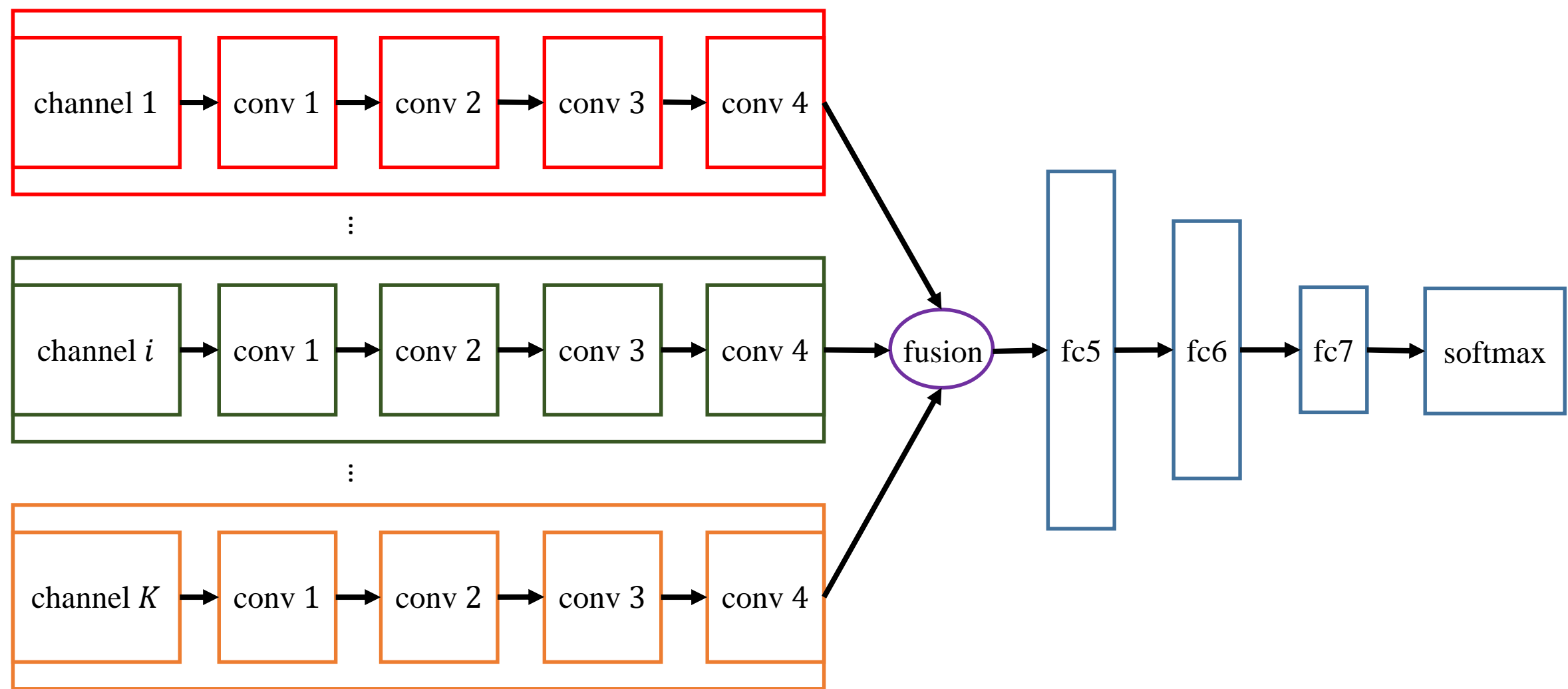

Supplement: Supplementary file 1 — LaTeX Supplementary File [file 41598_2018_37387_MOESM1_ESM.zip › Fig/multi-source_vivid.pdf]

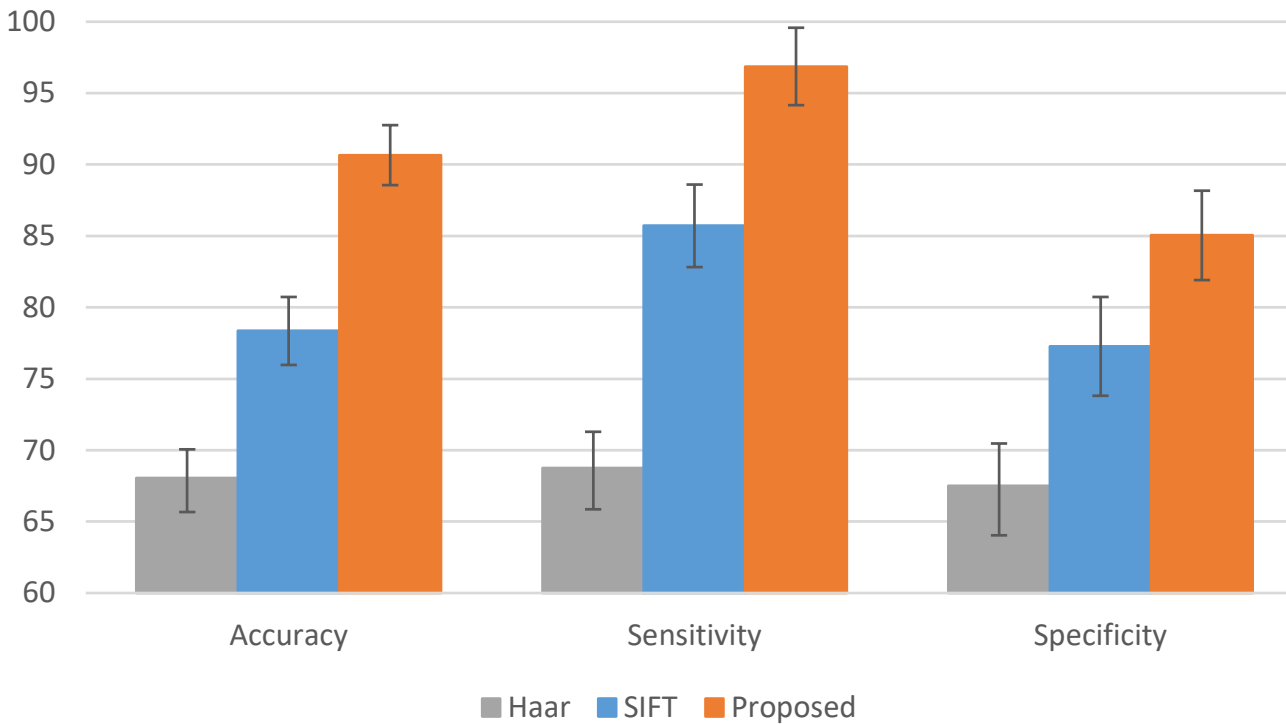

Supplement: Supplementary file 1 — LaTeX Supplementary File [file 41598_2018_37387_MOESM1_ESM.zip › Fig/supervisedComparison.pdf]

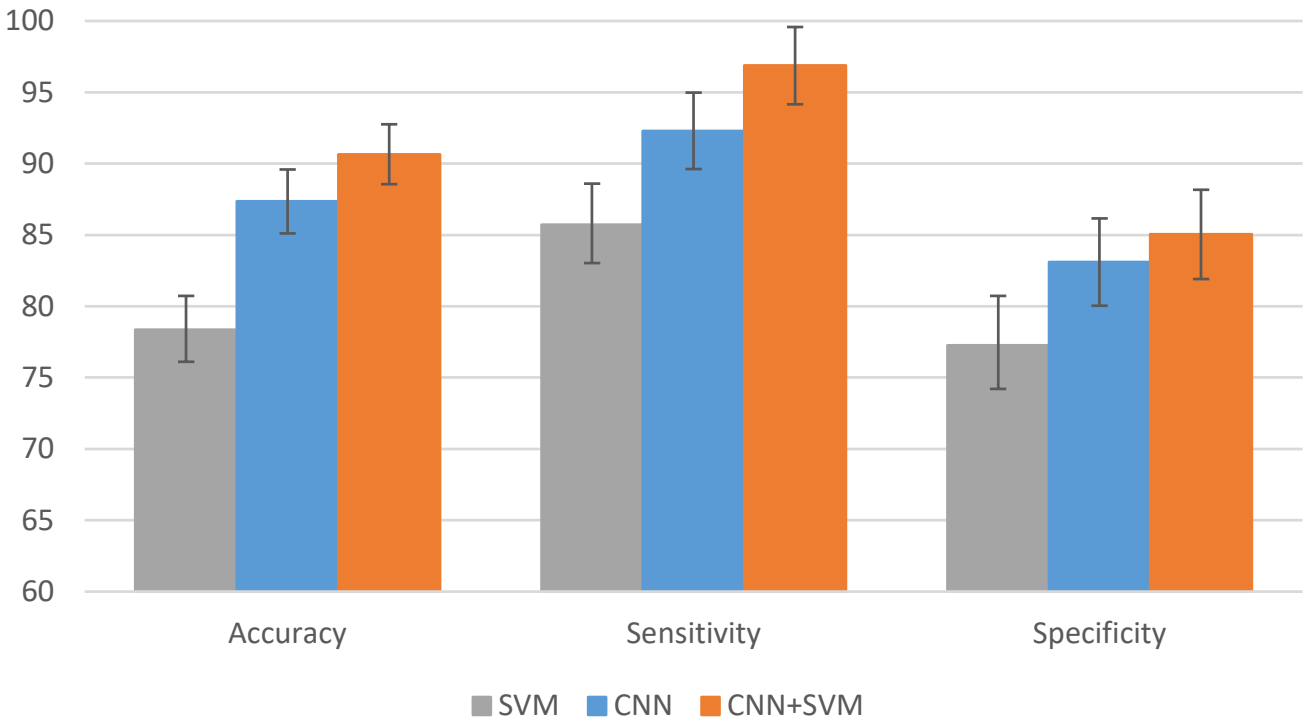

Supplement: Supplementary file 1 — LaTeX Supplementary File [file 41598_2018_37387_MOESM1_ESM.zip › Fig/svm_cnn_cnn-svm.pdf]
